# *Achnatherum inebrians* Bacterial Communities Associated with *Epichloë gansuensis* Endophyte Infection Under Low-Concentration Urea Treatment: Links to Plant Growth and Root Metabolite

**DOI:** 10.3390/microorganisms13071493

**Published:** 2025-06-26

**Authors:** Yuanyuan Jin, Zhenjiang Chen, Kamran Malik, Chunjie Li

**Affiliations:** State Key Laboratory of Herbage Improvement and Grassland Agro-Ecosystems, Key Laboratory of Grassland Livestock Industry Innovation, Ministry of Agriculture and Rural Affairs, Engineering Research Center of Grassland Industry, Ministry of Education, Gansu Tech Innovation Centre of Western China Grassland Industry, Centre for Grassland Microbiome, College of Pastoral Agriculture Science and Technology, Lanzhou University, Lanzhou 730000, China; jinyy@lzu.edu.cn (Y.J.); malik@lzu.edu.cn (K.M.)

**Keywords:** drunken horse grass, fungal endophyte, plant microbiome, root exudates, strains

## Abstract

Despite chemical exchange often serving as the first step in plant–microbe interactions, the specialized chemical metabolites produced by grass–*Epichloë* endophyte symbiosis as mediators of host growth, nutrient acquisition, and modulators of the rhizosphere community under low-nitrogen conditions are areas lacking in knowledge. In this study, we investigated the plant growth-promoting effects of the *Epichloë* endophyte strain and identified the growth of the *Epichloë* strain under different types of nitrogen source treatments. In addition to the in vitro test, we evaluated growth performance for *Epichloë* endophyte–infected plants (E+) and *Epichloë* endophyte–free plants (E−) in a pot trial under 0.01 mol/L urea treatment. Seedlings from E+ and E− groups were collected to analyze the plant bacterial microbiome and root metabolites. The *E. gansuensis* endophyte strain was found not to produce indoleacetic acid (IAA), pectinase, or contain ferritin. The nitrogenase gene, essential for nitrogen fixation, was also absent. These results suggest that *E. gansuensis* endophyte strains themselves do not contain attributes to promote plant growth. Concerning N fertilization, it was observed an increase in the colony diameter of *E. gansuensis* strain was observed only in the NO_3_^−^-N (NN) treatment, while inhibition was observed in the urea-N (UN) treatment. *E. gansuensis* endophyte symbiosis significantly increased tiller number and plant dry weight. Overall, our results suggest that the E+ plants had more root forks and greater average root diameter compared to E− plants under the UN treatment. In a pot experiment using UN, data from 16S rRNA amplicon sequencing revealed that *E. gansuensis* endophyte infection significantly altered the bacterial community composition in shoot and root, and significantly increased Shannon (*p* < 0.001) and Chao 1 (*p* < 0.01) indexes. The relative abundance of Acidobacteriota, Actinomycetota, Cyanobacteriota, Fibrobacterota, Myxococcota, and Patescibacteria in the shoot, and Cyanobacteriota, Pseudomonadota, and Verrucomicrobiota in the root were significantly increased by *E. gansuensis* endophyte infection. Similarly, *E. gansuensis* endophyte symbiosis shifted the metabolite composition of the host plants, with the E+ plants showing a higher number of metabolites than the E− plants. In addition, co-metabolism network analysis revealed that the positive relevance between exudates and microorganisms in the root of the E+ plants is higher than that of the E− plants. These findings provide valuable insights into the knowledge of the effects of the symbiotic relationship between host plants and *Epichloë* endophyte on interspecific interactions of plant microbiome, beneficial for harnessing endophytic symbiosis, promoting plant growth.

## 1. Introduction

Approximately 20–30% of all grass species are estimated to host systemic, foliar *Epichloë* fungal endophytes [1], which makes these associations of wide interest for the study of plant–fungal interactions. Fungal endophytes are expected to be obligate mutualists that obtain shelter and nutrition from host plants in exchange for either altering their physiology or producing alkaloids [2]. Extensive studies have confirmed that *Epichloë* endophyte infection increases host plant tolerance to biotic stresses (eg., insects, diseases, and nematodes) [3,4] and abiotic stresses (drought, salt, and cold) [5,6]. However, some studies have shown negative interactions in grass–endophyte symbiosis under certain conditions [7]. Previous studies on grass–*Epichloë* endophyte interactions have concluded that the degree of mutual benefit is conditional upon environmental factors, such as nutrient availability [8,9]. Differential nitrogen supply has different effects on endophyte and alkaloid concentration, and plant growth [10,11]. Understanding the interactions between *Epichloë* endophytes and their environment requires experiments that integrate variations in endophyte strains, grass species, and environmental factors. However, to the best of our knowledge, a comprehensive study on grass–*Epichloë* endophyte symbiosis remains lacking. *Achnatherum inebrians* (Drunken horse grass) is a perennial C4 grass with remarkable ecological adaptability; it is a toxic weed in degraded grasslands of northwest China [12] and is cultivated as a bird-repelling turfgrass that thrives in both agricultural soils and natural grasslands [13,14]. Subsequent studies revealed that the toxicity of *A. inebrians* plants is caused by infection with the asexual endophyte *Epichloë gansuensis* or *E. inebrians* endophytes [15]. The *E. gansuensis*/*E. inebrians* endophyte–*A. inebrians* symbiont is known to produce two alkaloids, ergine and ergonovine, which are toxic to mammalian herbivores and are considered the primary causes of *A. inebrians* toxicosis [12,16]. Furthermore, infection by *E. gansuensis* and *E. inebrians* endophytes has been reported to provide various benefits to *A. inebrians.* These advantages include enhanced tolerance to insect pests [17], pathogenic fungi [18,19], heavy metals [20], low temperature [21], drought [22], low N and low P [23,24]. These benefits have contributed to the expanded distribution of *A. inebrians*. *Epichloë* endophyte infection also significantly altered the community composition and increased diversity of bacteria and fungi in the phyllosphere and seeds of *A. inebrians* under normal growth conditions [25,26]. However, surprisingly few controlled studies have examined the effects of stressful environment factors on the plant microbiome in grass–*Epichloë* endophyte associations [27,28]. As a result, our ability to predict the consequences of the environmentally mediated the effects of the *Epichloë* endophyte on plant-associated microorganisms remains limited. In particular, few studies have explored the interactions between N availability and foliar *Epichloë* endophyte infection in shaping the plant microbiome [29].

In addition to their well-known effects on host plants, the *E. gansuensis* or *E. inebrians* endophytes have recently been shown to also alter the underground activities of host plants [30,31]. The *E. gansuensis* endophyte infection significantly enhanced the N content of host plants, and affected nutrient ratios of *A. inebrians* probably by increasing rhizosphere soil bacterial diversity and altering rhizosphere bacterial community structure during three different seasons [32]. Jin et al. [33] showed significant differences in the rhizosphere microbial communities involved in nitrification and denitrification among different ecotypes of *A. inebrians-Epichloë* endophyte symbiont. Wang et al. [34] observed that P addition modulates the changes in the soil bacterial community composition and root exudate profiles, mediated by the *E. gansuensis* endophyte. The mechanisms underlying the effects on the rhizosphere microbiome are largely unknown; these effects are likely directly due to the root exudates of host plant-associated *Epichloë* endophytes, or indirectly due to the presence of endophytes in litter [35,36]. 

In this study, we carried out an in vitro experiment designed to assess the plant growth-promoting effects of an *Epichloë* endophyte strain, and analyzed the growth of this *Epichloë* endophyte strain under different types of N (NH_4_^+^-N, NO_3_^−^-N and Urea-N) treatments. In addition to in vitro tests, integrated microbiome and metabolome data, and studied and compared the growth performance, plant microbiomes, and root metabolism of *Epichloë* endophyte–infected (E+) and *Epichloë* endophyte–free (E−) plants in a pot trial under low nitrogen conditions. The aims of this study were to (1) confirm whether the *E. gansuensis* endophyte strains themselves possess growth-promoting effects; (2) demonstrate the effects of different N types on the growth of the *Epichloë* endophyte strain; (3) delineate the variation patterns of the microbiome and metabolome between E+ and E− plants under low nitrogen conditions; (4) demonstrate the complexity of microbial interactions whether depend on *Epichloë* endophyte infection; and (5) explore the roles played by the root exudates of E+ plants on root microbial composition and furnish functional-level evidence for a deeper understanding of *E. gansuensis* positive effect. This study provides novel insights into understanding the symbiotic relationship between host plants and *Epichloë* endophyte on the stress resistance process in plants.

## 2. Materials and Methods

### 2.1. Plant Material

*Achnatherum inebrians* seeds from a Tianzhu ecotype whose mature reproductive tillers were originally grown in field environments in Gansu Province, China (Tianzhu, N: 36.97; E: 103.77; H: 2940 m) were used for this experiment. *Epichloë* endophyte infection in seeds and leaf sheaths was examined microscopically via the staining method described by [37]. E− seeds were obtained by killing endophyte viability through treatment with a 100-fold dilution of thiophanate–methyl (Jiangsu Rotam Chemistry Co., Ltd., Suzhou, China) in 2017. To eliminate potential negative effects of the fungicide on the health of *A. inebrians* plants, the treated seeds were carefully rinsed with water. The E+ and E− plants were cultivated in the field, and seeds were collected annually. Staining, microscopic examination, and PCR analyses using the following an *Epichloë*–specific PCR primer pair (tub2-exon 1d-1: GAGAAAATGCGTGAGATTGT; tub2-exon 4u-2: GTTTCGTCCGAGTTCTCGAC) [38] was used for screening–seeds. This ensures that the E− seeds were free of *Epichloë* endophytes. Seeds collected in July 2022 were stored at 4 °C to preserve endophyte viability. For the experiment, two hundred E+ and two hundred E− seeds were surface–disinfected with water: sodium hypochlorite (1:1 *v*/*v*) and then germinated on seedling-raising plates with sterile vermiculite in a constant-temperature greenhouse (22 °C).

### 2.2. Epichloë Endophyte Strain Isolation

Two months after germination, some E+ seedlings were transferred to the laboratory, washed with distilled water, and cut into small pieces. The E+ seeds and pieces were surface sterilized using 75% ethanol for 30 s and 1.0% NaOCl for 1 min, followed by thorough rinsing with sterilized water 5 times. *Epichloë* endophytes were isolated from sterilized seeds and seedlings of *A. inebrians* placed on potato dextrose agar (PDA), as described by [39]. From 10 to 30 days after incubation, the *Epichloë* endophyte grew and was isolated as a pure culture on fresh PDA medium through three rounds of purification.

### 2.3. Epichloë Endophyte Strain Identification

Fungal DNA was extracted using the D3195-01 HP Fungal DNA Mini Kit (Omega Biotek Inc., Norcross, GA, USA) following the manufacturer’s instructions. Fungal DNA was amplified on a CFX 96 TM Real-Time PCR Detection System (Bio Rad, Hercules, CA, USA). The respective reaction mixtures were composed, and the PCR conditions are shown in Table S1. Each qPCR mixture was prepared in a total volume of 25 μL, consisting of 12.5 µL of 2X Taq Master Mix, 9.5 µL of ddH_2_O, 2.0 μL of forward and reverse primers, and 1 µL of fungal DNA template. Triplicate independent PCRs were conducted for each DNA sample. The gel-purified PCR products were sequenced bidirectionally by Shenggong Bioengineering (Shanghai, China) Co., Ltd. and bidirectionally spliced. Basic Local Alignment Search Tool (BLAST v2.16.0) comparisons of the sequences were performed using the NCBI database (https://www.ncbi.nlm.nih.gov/, accessed on 20 April 2024), and the sequences were matched to published ITS for homologous identification.

### 2.4. Plant Growth-Promoting Effects of Epichloë Endophyte Strain

To evaluate the growth-promoting effects of the *Epichloë* endophyte strain, the concentration of indole acetic acid (IAA) was determined via the spectrophotometric method [40], the pectinase concentration was determined via DNS colorimetry [41], the ferritin carrier concentration was determined via colorimetry [42], and the absolute abundance of the *nifH* gene was determined via qPCR [43].

### 2.5. Experiment 1: Effects of Nitrogen Fertilizers on Epichloë Endophyte Strains

To study the effects of different nitrogen sources (NH_4_⁺-N, NO_3_⁻-N, and urea-N) on the growth of the *Epichloë* endophyte strain, basal culture media were prepared using 200 g of peeled potato (*Solanum tuberosum*), 20 g of glucose, 18 g of agar (liquid medium without agar) and 1000 mL water. Urea (10.72 g), NH_4_Cl (11.12 g), and NaNO_3_ (17.86 g) were added to the basal medium to create media with different nitrogen sources, and N content was 0.15 mol/L, 0.1 mol/L, and 0.1 mol/L. Solid and liquid PDA medium without N was used as a control. The solid medium was dispensed into 90 mm diameter Petri dishes, with approximately 30 mL per dish. After the Petri dishes had cooled, a layer of breathable film (cellophane) was placed on top of the medium in each dish. The liquid medium was transferred to a 200 mL triangular flask, with approximately 50 mL per flask. The experiment was conducted with five replicates for each nitrogen fertilizer treatment. The *Epichloë* endophyte strain was tested for its ability to utilize organic and inorganic nitrogen sources in both solid and liquid media. The *Epichloë* endophyte strain was incubated in liquid medium at 25 °C for 10 days with shaking at 150× *g*. The colony diameter of the fungal endophytes on solid media was measured using Vernier calipers, whereas the mycelial concentration of the fungal endophytes in liquid media was measured using the drying and weighing method.

### 2.6. Experiment 2: Effects of Urea on the Achnatherum Inebrians–Epichloë Endophyte Symbiont

#### 2.6.1. Experimental Design

On the basis of the results of experiment 1, urea was selected as the nitrogen source to study the effects of low-nitrogen treatment on the growth, bacterial microorganisms, and root metabolites of the *A. inebrians*–*Epichloë* endophyte symbiont. Our previous study revealed that *Epichloë* endophyte infection significantly promoted the nitrogen utilization efficiency of host plants under 0.1 mmol/L low-nitrogen treatment [29,44]. Therefore, we chose 0.1 mmol/L as the stress concentration for urea. After germination for 2 months, 10 cm-tall seedlings were transplanted into hydroponic boxes measuring 40 cm in length, 26 cm in width, and 25 cm in height. Each box, which contained six seedlings, was filled with 400 mL of modified ½-strength Hoagland’s nutrient solution (urea as a nitrogen source). A pot experiment with a completely randomized design with a factorial arrangement of treatments was established in an artificial greenhouse. The treatments included a single genotype of *A. inebrians* plants with two levels of endophyte infection (E+ and E−) and one nitrogen level. Each treatment was replicated four times. During the 63-day growth period, E+ and E− plants were cultivated at average day and night temperatures of 25 ± 2 °C and 18 ± 2 °C, respectively, with a 16 h: 8 h, light: dark photoperiod (c. 600 μmol m^−2^. s^−1^). Plant height and root length were measured using a ruler before harvest. Leaf and root tissues from E+ and E− plants (*n* = 6) subjected to low-nitrogen treatment were collected after 63 days of growth. Six plants were analyzed per treatment group, totaling seventy-two plants. The roots of E+ and E− plants were washed with deionized water and transferred to aluminum foil-wrapped conical flasks filled with low-nitrogen nutrient solution [45]. E+ and E− plants were incubated under a normal photoperiod (16 h light/8 h dark) at 26 °C for 7 d, after which the culture liquid was collected for metabolite profiling by filtration through a 0.22 μm membrane. The total length, diameter, volume, surface area, tip, and fork number of the roots were analyzed using a Perfection V550 scanner (Epson America Inc., Long Beach, CA, USA).

#### 2.6.2. Nutrient Content and Dry Weight Measurements of Shoots and Roots

The total carbon content in the shoots and roots of the E+ and E− plants was determined by using a FlashEA 1112 series CHNS/O analyzer (Thermo Fisher, Waltham, MA, USA). Shoots and roots from the E+ and E− samples were digested with H_2_SO_4_ solution and a catalyst (CuSO_4_:K_2_SO_4_: 1:10 mixture) at 420 °C for 1 h. The concentrations of total nitrogen (N) and total phosphorus (P) were then determined using an injection system (FIAstar 5000 Analyzer, Foss, Hillerød, Denmark). A constant weight was achieved by oven drying the samples at 80 °C for 48 h, and the dry weight was determined with an electronic balance.

#### 2.6.3. DNA Extraction and Sequencing

Total DNA extraction from the shoots and roots of E+ and E− plants (n = 6) was carried out using a modified CTAB DNA extraction method [46]. The concentration and purity of DNA were measured using a Micro NanoDrop ND-1000 UV–Vis Spectrophotometer (Nanodrop Technologies, Wilmington, DE, USA). The quality of DNA was evaluated via 1% agarose gel electrophoresis and then stored at −20 °C.

A total of 10 ng of DNA per sample was used to amplify the V3-V4 region of the 16S rRNA gene using an ABI GeneAmp^®^9700 PCR system. The amplification was performed using Trans Start FastPfu DNA Polymerase (TransGen AP221-02, TransGen Biotech, Beijing, China) and specific primers (338F: 5′-ACTCCTACGGGAGGCAGCAG-3′ and 806R: 5′-GGACTACHVGGGTWTCTAAT-3′) [47]. The PCR products from the same samples were pooled and analyzed via 2% agarose gel electrophoresis. The PCR products were recovered from the gel using the AxyPrepDNA Gel Recovery Kit (Axygen-Biosciences, Union, CA, USA) and eluted with Tris-HCl. Four biological replicates with the highest DNA quality were screened for *16S rRNA* amplicon sequencing, which was performed on an Illumina MiSeq PE300 (Illumina Inc., San Diego, CA, USA) sequencing platform by Shanghai Majorbio Bio-pharm Technology Co., Ltd. (Shanghai, China) [43].

#### 2.6.4. Metabolite Extraction and UHPLC–MS/MS Analysis

Metabolite analysis was conducted following previously established protocols [48]. 400 µL of a methanol: acetonitrile (1:1, *v*/*v*) mixture was added to 100 µL of the root extraction mixture collected from the E+ and E− plants, respectively. The mixture was sonicated at 40 kHz for 30 min at 5 °C. Samples were incubated at −20 °C for 30 min to precipitate the proteins, followed by centrifugation at 13,000× *g* for 15 min at 4 °C. The resulting supernatants were transferred to fresh vials and dried under a gentle stream of nitrogen gas. After drying, the metabolites were reconstituted in 100 µL of an acetonitrile: water (1:1, *v*/*v*) mixture by brief sonication in a water bath maintained at 5 °C. The reconstituted samples were then centrifuged at 13,000× *g* for 15 min at 4 °C. The supernatants were stored at −80 °C until ultrahigh-performance liquid chromatography–tandem mass spectrometry (UHPLC–MS/MS) analysis (Thermo Fisher, Waltham, MA, USA).

To investigate root metabolic N responses in the E+ plants, UHPLC–MS/MS analysis was used to determine the main root exudates in the E+ and E− plants. Chromatographic separation of the metabolites was performed using a UHPLC system coupled with an electrospray ionization (ESI) source operating in either positive (POS) or negative (NEG) ion mode. The analysis followed a previously established protocol [49]. Briefly, metabolite separation was performed by injecting a 2 μL sample onto a Waters HSS T3 column (100 mm × 2.1 mm, 1.8 μm). The mobile phase consisted of solvent A (water with 0.1% formic acid: acetonitrile, 95:5, *v*/*v*) and solvent B (acetonitrile: isopropanol: water with 0.1% formic acid, 47.5:47.5:5, *v*/*v*). The separation was carried out at a flow rate of 0.4 mL/min. Quality control (QC) samples and reference samples were injected every ten samples during the analytical run to monitor and evaluate the stability of the LC–MS system.

#### 2.6.5. LC–MS Data Processing and Annotation

Following UHPLC–MS/MS analyses, the data acquired through data-dependent MS/MS acquisition (DDA) were processed using Progenesis QI 3.0 (Nonlinear Dynamics, Waters, USA) for data preprocessing. The processing steps included baseline filtering, retention time (RT) alignment, lock-in mass correction, peak recognition and integration (excluding low-quality peaks), adduct grouping, and deconvolution. The parameters used were as follows: peak picking with the automatic sensitivity method (default settings) and an RT range of 0.1–8.0 min. Annotated metabolites were identified on the basis of retention time (RT) and *m*/*z* information (designated as features). Prior to downstream statistical analyses, ion abundances were normalized to the internal standard telmisartan by sum, on the basis of four biological replicates. Subsequent multivariate analyses, including Venn analysis, principal component analysis (PCA), and partial least squares discriminant analysis (PLS–DA), were conducted using the Venn Diagram and ropls package (v1.20.0) in R [50]. Feature annotations were conducted using the Kyoto Encyclopedia of Genes and Genomes (KEGG, http://www.genome.jp/kegg/, accessed on 15 May 2024), Metlin (https://metlin.scripps.edu/, accessed on 15 May 2024), Human Metabolome (HMDB, http://www.hmdb.ca/, accessed on 15 May 2024), and Majorbio databases based on 10 ppm precursor mass tolerance, 95% isotope similarity, and 10 ppm fragment mass tolerance. To accurately evaluate the metabolic features of the root exudates between endophyte–infected plants and endophyte–free plants under urea treatment, 564 positive mode mass features (identified as RT: *m*/*z* ratio pairs) after data preprocessing were used for downstream statistical analysis.

#### 2.6.6. Mining of Differentially Abundant Metabolites

Multivariate statistical analysis was performed on the Majorbio Cloud Platform (https://cloud.majorbio.com, accessed on 1 June 2024). Orthogonal least partial squares discriminant analysis (OPLS–DA) was performed using the ROPLS package (v 1.6.2) in R to identify metabolic changes between the E+ and E− samples. The stability and reliability of the model were evaluated using 7-fold cross-validation and response permutation testing. Differentially expressed metabolites were identified on the basis of the variable importance in the projection (VIP) score and the following parameters: VIP > 1; *p* value of Student’s *t* test < 0.05; and fold change (FC) < 1 or FC > 1, using the default settings. The biochemical pathways of the significantly different metabolites were mapped with metabolic enrichment and pathway analysis using the KEGG database. Significantly enriched pathways were identified using Fisher’s exact test (*p* < 0.05) implemented in the Stats 2.0 package in R and the SciPy package (v 1.11.4) (https://docs.scipy.org/doc/scipy/, accessed on 1 June 2024).

### 2.7. Bioinformatics Analysis

The raw sequence reads obtained via Illumina sequencing were first assembled based on overlap relationships and then quality-filtered and trimmed using fastp v0.19.6 with adapter autodetection and the default parameters [51]. Low-quality reads (length < 50 bp or with a quality value < 20 or having N bases were removed. The quality-filtered reads (34,000–43,000 sequences) were processed using the QIIME 2.0 pipeline on the free online platform of Majorbio Cloud (www.majorbio.com, accessed on 5 June 2024). The optimized reads were further processed using sequence noise reduction methods (DADA2) to generate representative amplicon sequence variants (ASV) sequences and their corresponding abundance information. To determine the species classification for each ASV, the Silva database on the QIIME (from Latin *Silva*, forest, http://www.arb-silva.de, accessed on 6 June 2024) on the Qiime 2.0 platform (https://qiime2.org, accessed on 6 June 2024) was used to analyze the taxonomy of the ASV representative sequences.

The number of ASVs shared and unique between the shoots and roots of the E+ and E− plants was analyzed using a Venn diagram generated by VennDiagram package (v1.7.3) in R (v. 3.3.1). Relative abundances at the bacterial class taxonomic rank were analyzed and visualized for the 30 most abundant phyla using bar a chart. The Wilcoxon rank-sum test was used to detect species differing in abundance in the microbial community between the E+ and E− plants under urea treatment. The Shannon and Chao1 diversity indices were calculated via Student’s *t* test by Mothur v1.30.2 (https://mothur.org/wiki/download_mothur/, accessed on 8 June 2024) software in R. Principal coordinate analysis (PCoA) was performed with the ordinate phyloseq R function by first transforming relative abundances into a Bray–Curtis dissimilarity matrix using the vegdist function and differences in species composition between the E+ and E− samples were calculated using the Adonis function within the vegan v3.3.1 R package.

### 2.8. Statistical Analyses

The statistically significant differences in plant biomass, plant nutrients, and bacterial community diversity between E+ and E− plants were evaluated by an independent sample *t*-test at a threshold *p*-value  <  0.05. A significant effect of different N sources on colony diameter and mycelial concentration of *E. gansuensis* endophyte strain was analyzed with a one–way ANOVA (*p* < 0.05). All analyses of variance (ANOVA) were performed using SPSS statistical software (Version 20.0, Inc., Chicago, IL, USA).

### 2.9. Analysis of the Microbial Co-Occurrence Networks in the Shoot, Root, and Root Microbiota–Metabolite Interactions

We elucidated the co-occurrence correlations between metabolomics and microbiomics using network analysis under U-N treatment, focusing on differential root metabolites and root bacterial microbes at the genus level on the basis of Spearman’s rank correlation coefficient. Spearman’s rank correlation coefficient was calculated among microorganisms and between microbes and metabolites. The obtained numerical matrix was used for a hierarchical clustering with complete linkages and visualized using the heatmap function within the Heatmap v3.3.1 R package. To investigate how microbial interactions differ between the shoots and roots of *A. inebrians*, microbial co-occurrence networks were constructed using the “NETWORKX v1.11” python package on the basis of significant pairwise Spearman’s correlations between bacterial microbial taxa in the shoots and roots of the E+ and E− plants [52]. The network complexity was then compared between the shoots and roots as well as between the E+ and E− plants. The nodes and edges in the network represent bacterial ASVs and significant interactions between pairs of ASVs; for a cumulative degree distribution, see Supplemental Files S1 and S2. To identify metabolites that play a key role, we investigated the correlation between root bacteria and root exudates using a two-factor correlation network analysis. The ASVs with relative abundances of less than 0.01% in the E+ and E− samples were filtered out because they were poorly represented [53]. Only rank correlation coefficients with values greater than 0.7 or less than −0.7 and a statistically significant *p* value (*p* < 0.05) were considered valid correlations in the network. The bacterial networks in the shoots and roots were graphically visualized using Cytoscape (v.3.7.0). To assess the response of microbial interactions in the shoot and root, as well as root microbial–metabolite interactions under urea nitrogen supply, six network properties were quantified: network diameter, degree, closeness centrality, betweenness centrality, degree centrality, and degree distribution. These metrics have been commonly used in previous studies to evaluate microbial community stability [54].

## 3. Results

### 3.1. Impact of Different N on the Growth of the Epichloë Strain

**Figure 1:**
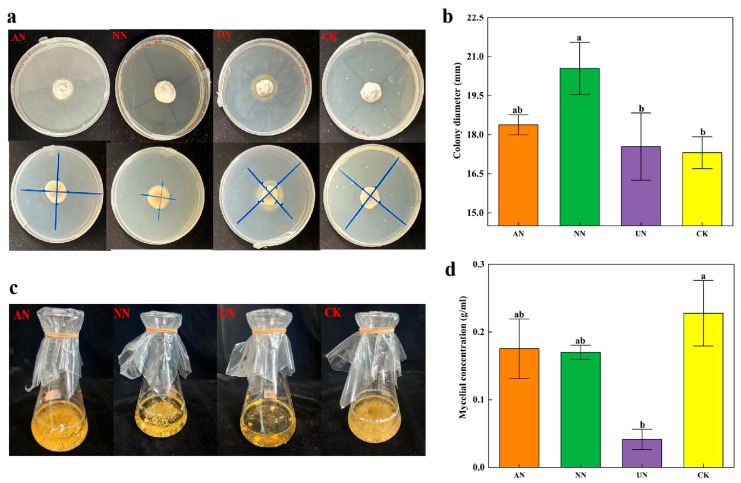
The effects of ammonium nitrogen (AN), nitrate nitrogen (NN), and urea (UN) additions on (**a**,**b**) colony diameter in solid culture medium and (**c**,**d**) mycelial concentration in liquid culture medium of *Epichloë* endophyte strains. CK: solid and liquid PDA medium. Different lowercase letters indicate significant differences.

The *Epichloë* endophyte strain was identified as the *E. gansuensis* endophyte by DNA sequencing and nucleotide comparisons of the ITS on the NCBI website (Table S2 and Figure S1a). This strain did not produce indoleacetic acid, pectinase, or ferritin carriers and did not possess the nitrogenase gene required for nitrogen fixation or the potential for nitrogen fixation (Table S2 and Figure S1b). Nitrate nitrogen (NN) was the only treatment that significantly increased colony diameter (*p* < 0.01) compared to the control (CK, PDA medium), whereas urea nitrogen (UN)significantly inhibited growth (Figure 1b). An inhibition zone formed around the colony of the endophyte strain under urea treatment (Figure 1a,c). In liquid culture medium, urea treatment significantly inhibited the colony-forming ability of the *E. gansuensis* endophyte strain (Figure 1d).

### 3.2. Urea Modulation of the Stimulatory Effect of the Epichloë Endophyte on Root Morphology and Plant Weight

**Figure 2:**
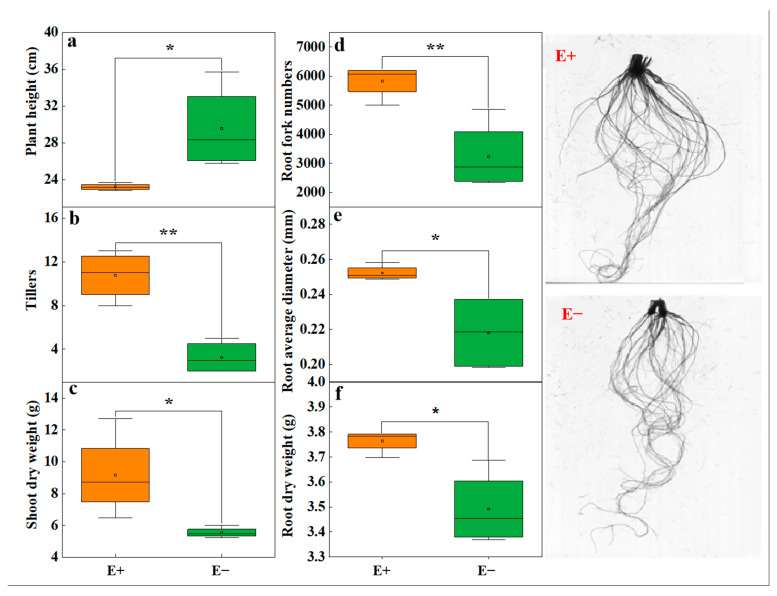
The effects of *Epichloë* endophyte on (**a**) plant weight, (**b**) tillers, (**c**) leaf dry weight, (**d**) root fork number, (**e**) root average diameter, and (**f**) root dry weight between *Epichloë* endophyte–infected (E+) plants and *Epichloë* endophyte–free (E−) plants under low-concentration urea treatment. The data were considered significant only if the *p* < 0.05 (* indicates *p* < 0.05 and ** *p* < 0.01).

On the basis of the results of experiment 1, a pot experiment was conducted to evaluate whether urea also had an inhibitory effect on the growth of the *A. inebrians*–*E. gansuensis* endophyte symbiont. *E. gansuensis* endophyte symbiosis significantly increased the total C content in the shoots and roots of host plants, but had no significant effect on the total nitrogen content (Figure S2). The *Epichloë* endophyte infection significantly reduced the plant height (Figure 2a), but significantly increased the tiller number (Figure 2b) and dry weight of the leaves (Figure 1c). Independent sample *t* tests suggested that the E+ plants presented greater root fork numbers and greater root dry weights than the E− plants (Figure 2d,f). The root average diameter of the E+ plants was greater than that of the E− plants under urea treatment (Figure 2e).

### 3.3. Effects of Urea on the Composition and Diversity of the Bacterial Community in the Shoots and Roots of the E+ and E− Plants

**Figure 3:**
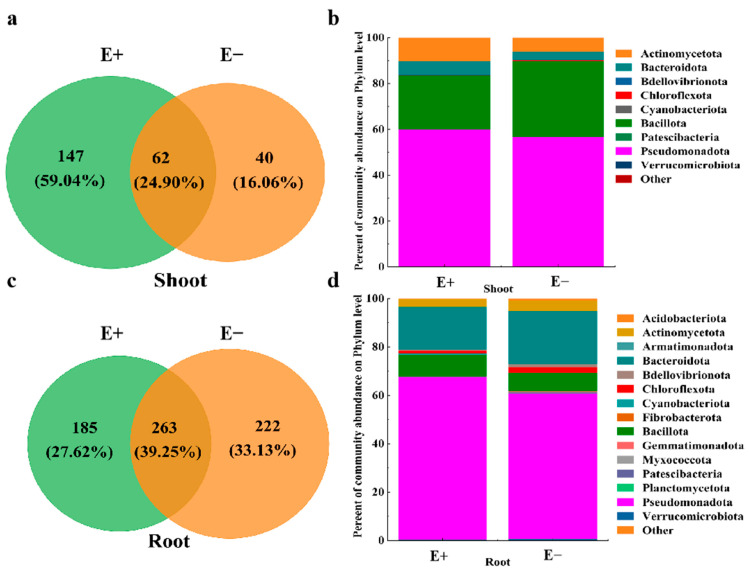
Venn diagram showing the number of shared and unique ASVs, and bar plots showing bacterial microbial community composition in (**a**,**b**) shoot and (**c**,**d**) root between *Epichloë* endophyte–infected (E+) and *Epichloë* endophyte–free (E–) plants under low-concentration urea treatment, separately.

The numbers of unique ASVs of shoot bacteria in the E+ plants was 147, which was greater than that of shoot bacteria in the E− plants (40) (Figure 3a), whereas the number of unique ASVs in the E+ roots (185) was lower than that in the E− roots (222) (Figure 3c). Pseudomonadota (60.04% and 56.61%), Bacillota (23.39% and 33.23%) and Actinomycetota (11.31% and 6.14%) accounted for more than 91.0% of the total bacterial sequences (Figure 3b). For the root bacterial community, the dominant phyla between the E+ and E− plants were Pseudomonadota (67.60% and 60.93%), Bacteroidota (17.56% and 21.90%) and Bacillota (9.14% and 8.36%) (Figure 3d). In addition, Chloroflexota and Actinomycetota were also present in the two samples with relative abundances between 1.64% and 4.38% (Figure 3d).

**Figure 4:**
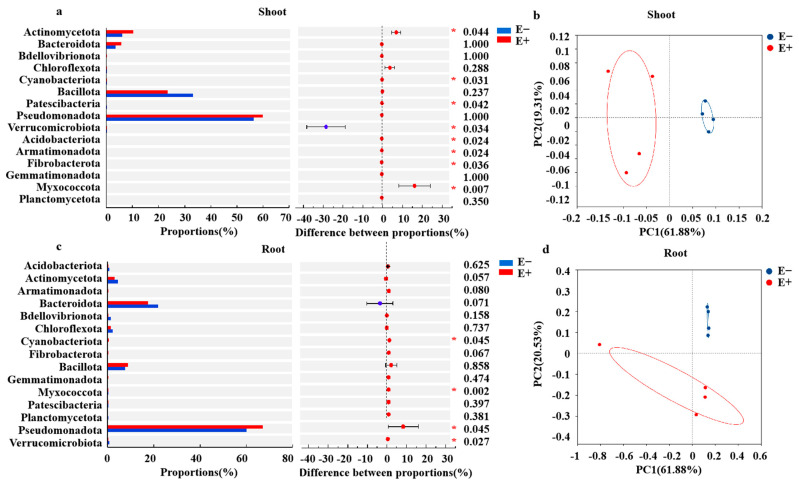
Wilcoxon rank sum test analyzes bacterial communities that differ significantly in (**a**) shoot and (**c**) root between *Epichloë* endophyte–infected (E+) and *Epichloë* endophyte–free (E–) plants under low-concentration urea treatment. *X*-axis: difference between proportions of bacterial communities at different phyla; Different colored boxes indicate the E+ and E− plants; *Y*-axis represents the average relative abundance of bacterial phyla between the E+ and E− plants. Principal co-ordinates analysis (PCoA) plot based on Euclidean distances to detect similarity or difference structure of the bacterial community composition in shoot (**b**) and root (**d**) of *Achnatherum inebrians* infected and uninfected with *Epichloë gansuensis* endophyte under low-concentration urea treatment. Red * represent significant differences between E+ and E− plants at *p* < 0.05.

The relative abundances of Myxococcota, Fibrobacterota, Armatimonadota, Acidobacteriota, Patescibacteria, Cyanobacteriota, and Actinomycetota were significantly greater in E+ shoots than in E− shoots under urea treatment, but the relative abundance of Verrucomicrobiota in the E+ shoots was significantly lower than that in the E− shoots (Figure 4a). The *Epichloë* endophyte significantly increased the relative abundances of Verrucomicrobiota, Pseudomonadota, Myxococcota and Cyanobacteriota (Figure 4c). *Epichloë* endophyte infection significantly increased the Shannon and Chao1 indices of the shoot bacterial community (Figure S3a), but there was no obvious difference in the Shannon and Chao1 indices of the root bacterial community between the E+ and E− plants (Figure S3b). In addition, PCoA revealed that both the shoot and root bacterial community structures significantly differed (Anosim test) between the E+ and E− plants under urea treatment (Figure 4b,d), indicating that the *E. gansuensis* endophyte strongly affected the bacterial community structure, especially the shoot microbial community.

### 3.4. Epichloë Gansuensis Symbiosis Alters the Metabolite Composition of the Host Plant

**Figure 5:**
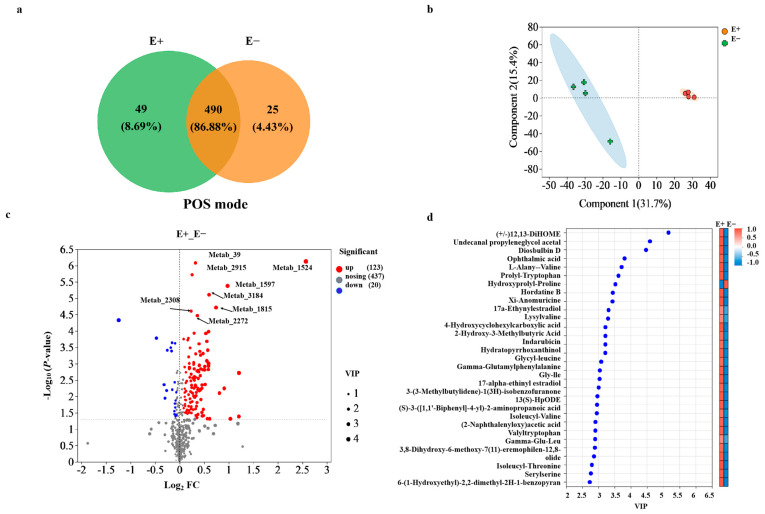
(**a**) Venn diagram showing the number of shared and unique metabolites between the E+ and E− plants under urea treatment. (**b**) Orthogonal partial least-squares discriminant analysis (OPLS-DA) plots showing differences in the composition of root exudates between the E+ and E− plants under low-concentration urea treatment. (**c**) Volcano plot visualizing up-regulated, down-regulated, and nosing metabolites between the E+ and E− plants under urea treatment. The *X*-axis values indicate fold changes in different metabolites between the E+ and E− plants, i.e., log_2_FC. The *Y*-axis values indicate the statistical test value for expression change in difference metabolites, i.e., −log_10_(*p*-value), with higher values indicating the greater the difference in metabolites between the E+ and E− plants. Each point represents a metabolite, and the point size indicates the Vip value. The red dots on the right and the blue dots on the left t are metabolites with up-regulated and down-regulated expression differences, respectively, and the closer the dots are to the upper right and left indicate metabolites with more significant differences between the E+ and E− plants. (**d**) VIP bar plot and clustered heat-map showing the top 30 metabolites with the greatest differences between the E+ and E− plants under low-concentration urea treatment.

PCA showed distinct separation in various metabolites between the E+ and E− plants under urea treatment (Figure S4). Aligned with the shoot microbial changes, biostatistical analysis of the untargeted metabolomic data by Venn analysis revealed that the E+ plants had a greater number of metabolites than did the E− plants under pos mode (Figure 5a). The metabolite profiles in the roots significantly differed between the E+ and E− plants according to OPLS-DA analysis (Figure 5b). The relative abundance of these compounds (123) was significantly higher in the E+ plants under urea treatment (Figure 5c). Furthermore, 189 compounds with identified features were found in the roots of the E+ plants and E− plants via KEGG analysis. Most compounds showing differences in accumulation in the E+ and E− plants were classified as organic acids and derivatives, organoheterocyclic compounds, and lipids and lipid-like molecules based on KEGG database searches for each feature (Supplemental File S3). In addition to the general classes of carbohydrates, acids, or alcohols metabolites, a few special features and unidentified compounds were enriched in the E+ and E− plants (Supplemental File S3). Among 30 metabolites with the greatest differences between the E+ and E− plants, with the exception of hydroxyprolyl-proline, the relative abundance was significantly higher in the roots of the E+ plants than in the roots of the E− plants (Figure 5d). *E. gansuensis* endophyte infection significantly increased the relative abundance of special compounds (e.g., (+/−)12, 13-DiHOME, undecanal, propyleneglycol acetal and diosbulbin D) in root exudates of *A. inebrians* (Figure 5d), but the identification of these compounds was tentative, and further studies are needed to determine.

The main metabolic pathways associated with the metabolites included amino acid metabolism (14 compounds), biosynthesis of other secondary metabolites (8 compounds), and lipid metabolism predominates (6 compounds) (Figure S5). KEGG topology analysis revealed that the metabolites in the roots of the E+ and E− plants were involved in purine metabolism (*p* = 0.001), tryptophan metabolism (*p* = 0.005), lysine biosynthesis (*p* = 0.011) and phenylalanine metabolism (*p* = 0.036), with tryptophan metabolism being the main pathway associated with the metabolites of the E+ plants metabolites (Figure S6).

### 3.5. Foliar Presence of the Epichloë Endophyte Increased the Shoot Microbial Co-Occurrence Network Complexity and Positive Interaction

**Figure 6:**
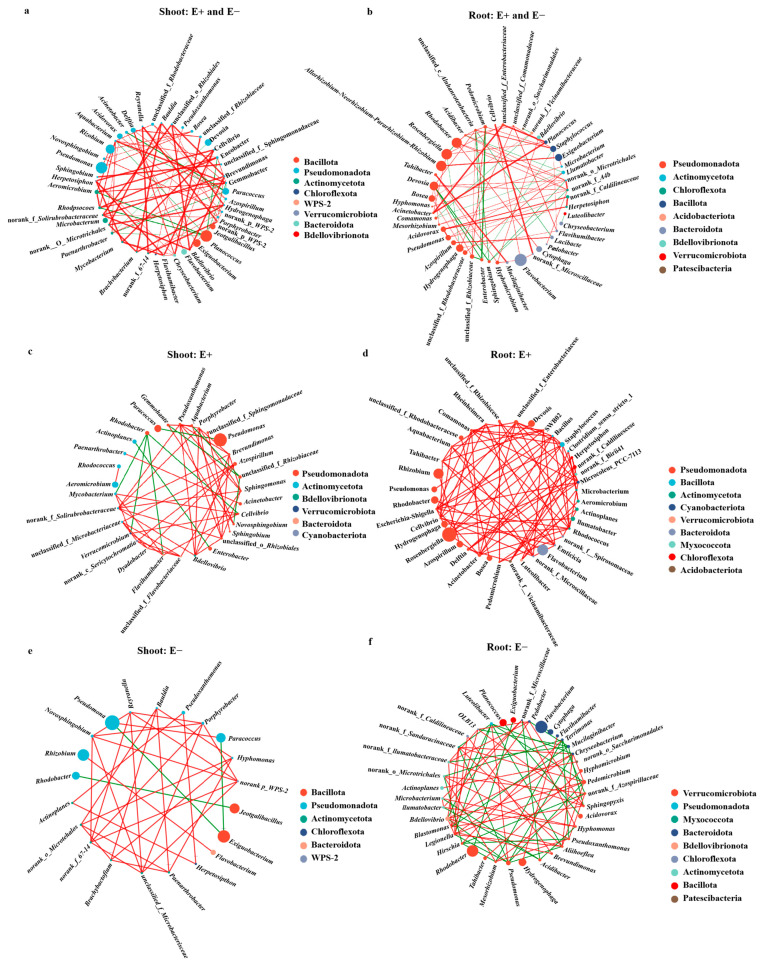
Co-occurrence networks of shoot and root bacterial communities based on a correlation analysis under low-concentration urea treatment: (**a**) the E+ and E− shoots; (**b**) the E+ and E− roots; (**c**) the E+ shoots; (**d**) the E− shoots; (**e**) the E+ shoots; (**f**) the E− roots. The red and green connecting lines indicate the positive and negative relationship between bacterial communities, respectively.

To investigate whether and how *E. gansuensis* endophyte infection affects the complexity and stability of molecular ecological networks, we constructed bacterial co-occurrence networks (Spearman’s correlation coefficient (ρ) was > 0.7 and *p*  <  0.05). The bacterial co-occurrence networks in shoot of *A. inebrians* plants consisted 45 nodes (genus) and 148 edges (Figure 6a), and the bacterial co-occurrence networks in shoot of the *A. inebrians* plants consisted 45 nodes (genus) and 148 edges in the shoots (Figure 6a), and 46 nodes with 146 edges in the roots (Figure 6b). For the shoot bacterial community, compared to E− plants, shoots of E+ plants samples had more nodes and edges, with higher connectivity and average degree (Supplemental Files S1 and S2, and Figure 6c,d). These more complex topological properties of bacterial networks in E+ plants represented stronger microbial interactions in shoots of *Epichloë* endophyte–infected plants. In the root bacterial network, infection with the *E. gansuensis* endophyte did not significantly increase the total number of nodes (39) or total number of edges (168) compared with those of the E− plants (40 nodes and 154 edges), but far more positive interconnections were observed in the roots of the E+ plants that in the roots of the E− plants under U-N treatment (Figure 6e,f).

### 3.6. Correlation Analysis Between Microbes and Metabolites in the Roots

**Figure 7:**
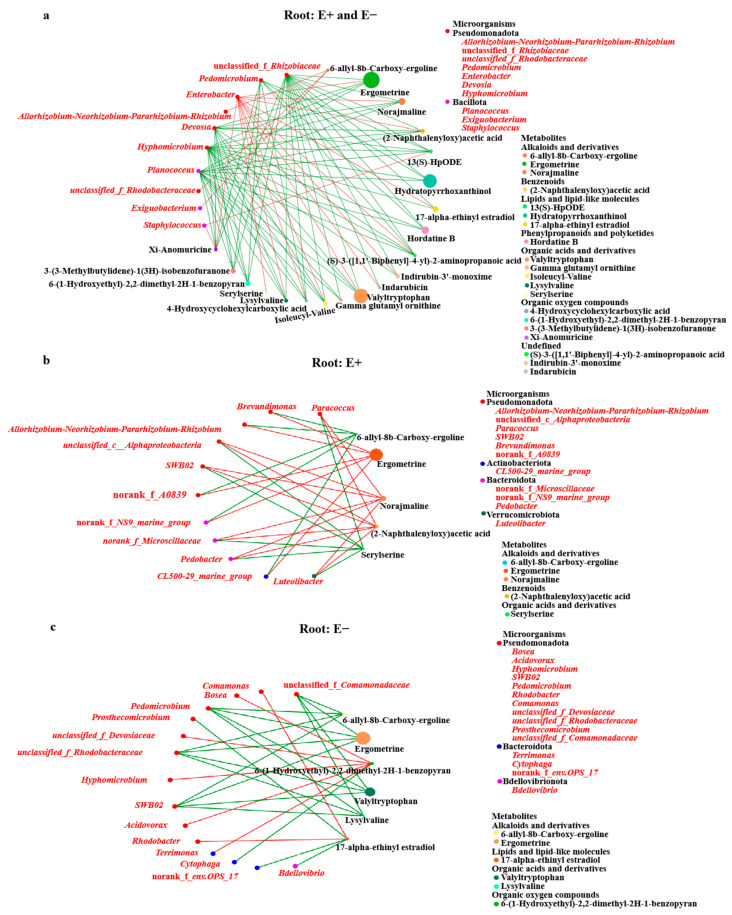
Co-metabolism network shows the associations between different metabolites and microorganisms: (**a**) the E+ and E− root compounds and bacterium; (**b**) the E+ root compounds and bacterium; (**c**) the E− root compounds and bacterium. Red/green lines: the positive/negative correlations between compounds and bacteria.

There were 84 significant (*p* < 0.05) correlations between 10 root bacterial microbes and 220 root metabolites present in the co-occurrence network (Figure 7a). There were 84 significant (*p* < 0.05) correlations between 10 root bacterial microbes and 220 root metabolites present in the co-occurrence network (Figure 7a). The co-metabolism network revealed that there was a significant positive correlation between *Enterobacter* (Pseudomonadota) and most root metabolites (Figure 7a). To further characterize the effects of *E. gansuensis* endophyte–mediated-root metabolites of *A. inebrians* by mediating on the plant root-associated microbiomes, we assessed the co-occurrence patterns of bacterial communities and metabolites between the E+ and E− plants. There were 28 significant (*p* < 0.05) correlations between 11 root bacterial microbes at the genus level and five root metabolites in the E+ plant network and 27 significant (*p* < 0.05) correlations between 15 microbes and six metabolites in the E− plant network (Figure 7b,c). Our results suggested that the complexity of the network constructed by *E. gansuensis* endophyte infection was greater. These bacteria were positively correlated with ergometrine, norajmaline and (2-naphthalenyloxy) acetic acid in the co-occurrence correlations between the metabolome and the microbiome of the E+ plants but were negatively associated with 6-allyl-8b-carboxy-ergoline and serylserine (Figure 7b). 6-(1-Hydroxyethyl)-2,2-dimethyl-2H-1-benzopyran had a positive correlation with other bacteria in the microbiome, with the exception of a negative correlation with *Cytophaga* in the co-occurrence correlations between the metabolome and the microbiome in the E− plants (Figure 7c). 6-Allyl-8b-carboxy-ergoline, ergometrine, valtyltryptophan and lysylvaline were negatively correlated with most bacteria in the microbiomes (Figure 7c).

## 4. Discussion

### 4.1. Urea Inhibited the Growth of the Epichloë Gansuensis Endophyte Strain

Symbiotic *Epichloë* endophytes have been reported to offer various benefits to their host plants; however, their effectiveness is influenced by environmental conditions and the specific genotypes of both the plant and the fungus itself [55]. In the present study, *A. inebrians* plants formed a symbiotic relationship with the *E. gansuensis* endophyte strain. However, the *E. gansuensis* endophyte strain was unable to grow on nitrogen-free agar media because of its inability to produce indole acetic acid and the absence of the *nifH* gene (Table S2). All alkaloids from the grass–*Epichloë* endophyte symbiont are nitrogen-containing organic compounds, with amino acids serving as the primary precursors in their biosynthetic pathway [56]. N fertilization influences the production of specific alkaloids in *Epichloë* endophyte–infected grasses [57]. To date, knowledge regarding the growth of *Epichloë* endophyte strains under different nitrogen treatments remains limited. The findings of the present study indicated that nitrate nitrogen significantly increased the growth of the endophyte strain, while urea negatively impacted endophyte growth by reducing the mycelial concentration of the *E. gansuensis* endophyte strain in liquid culture media and forming an inhibition zone in solid culture media (Figure 1). These results align with previous studies that indicate under in vitro conditions, the plant growth-promoting *Epichloë* endophyte and urea addition do not have synergistic effects [58]. This may occur because *E. gansuensis* endophyte strains themselves lacked the metabolic pathways required for urea nitrogen utilization. However, additional studies are needed to confirm the conjectures.

### 4.2. Plant Bacterial Communities Recruited by Epichloë Gansuensis

The phyllosphere and rhizosphere microbiota are well-known for their critical roles in promoting plant growth, suppressing disease, and enhancing resistance to biotic and abiotic stresses [59,60]. Extensive research on the microbiome of *A. inebrians* plants has revealed that the microbial community assemblies in the phyllosphere, seeds, roots, and rhizosphere are significantly influenced by *Epichloë* endophyte infection [25,26]. These results provided compelling evidence that *Epichloë* endophyte serves as one of the key drivers of plant microbial community dynamics, highlighting their pivotal role in facilitating host adaptation processes (9, 33). The current study demonstrated that the low-concentration urea treatment significantly altered the bacterial community composition of the shoots and roots of *A. inebrians* plants. These findings are consistent with those of previous studies by [29,61], which demonstrated that *A. inebrians* plants infected with the *E. gansuensis* endophyte hosted more complex shoot microbial communities compared to those of the E− plants under low concentrations of NH_4_NO_3_ and NH_4_^+^ treatments. These findings illustrate an amplified urea-responsive effect of the endophyte, which in turn conferred benefits to the host plants by increasing adaptability to stressful environments.

Our findings further demonstrated that the *Epichloë* endophyte significantly increased the relative abundance of oligotrophic bacteria (Acidobacteriota), phoD-harboring bacteria (Actinomycetota), Myxococcota, Fibrobacterota, Armatimonadota, Patescibacteria, and Cyanobacteriota. Notably, members of Myxococcota form multicellular structures (fruiting spores) to increase resistance to adverse environments during nutritional deficiency. Members of this class are known to prey on soil bacteria and fungi as a means to obtain energy and act in the degradation of complex organic matter, thus indirectly impacting carbon storage and the cycling of soil carbon pools [62]. Armatimonadetes play a role in soil carbon recycling by decomposing organic matter [63]. As suggested by previous studies, copiotrophic bacteria thrive under nutrient-rich conditions, whereas oligotrophic bacteria dominate in nutrient-poor environments because of their efficient nutrient utilization [29]. These results suggested that *Epichloë* endophyte infection might induce a mutual collaborative microbiome with different functions or oligotrophic bacteria to promote nutrient uptake by host plants.

Numerous studies have demonstrated that the alpha diversity of shoot and root microbiomes plays a critical role in regulating plant health, growth, and environmental adaptability [64]. The *Epichloë* endophyte significantly increased the α (Shannon and Chao1 indexes) and β (PCoA) diversity of shoot bacteria under urea treatment but had no significant effect on the root microbiome. This finding is inconsistent with our previous study results, which revealed that the *Epichloë* endophyte increased the α and β diversity of the shoot and root bacterial communities under low-concentration NH_4_^+^ treatment [29]. This discrepancy is possibly attributed to differences in the type of nitrogen fertilizer used.

These findings underscore the potential role of nitrogen availability as a selection driver in enabling foliar endophyte effects to shape bacterial communities. However, the magnitude of the effect differs significantly between organic (e.g., urea) and inorganic (e.g., nitrate) nitrogen sources. Further investigation of the multifaceted interplay between environmental factors (eg., nitrogen sources), *Epichloë* endophyte, and plant bacterial microbiota will provide deeper insights into the factors shaping holobiont resilience.

### 4.3. The Epichloë Gansuensis Endophyte Increases Shoot Microbial Network Complexity

Network analysis-based approaches have been widely applied in community assembly as effective tools for exploring the complex interaction networks [65]. In the present study, variations in bacterial interactions between shoots and roots in response to N treatment were explored through the construction of co-occurrence networks. The results indicated that N treatment decreased the number of positive network connections and weakened the interaction patterns of the co-occurrence networks in the bacterial communities in the roots compared with those in the shoots. These results are consistent with previous findings that the application of N and P fertilizers influences the complexity of bacterial microbial networks [61,66]. In a meta-community co-occurrence network, node–level topological features are employed to identify potentially important microbes, referred to as keystone taxa, and to uncover patterns of microbial co–occurrence [67]. This is indicated by the large differences in the number of nodes and edges of the shoot bacterial network, with high values of topological features, when the E+ networks are compared with the E− networks, which aligns with previous findings [61]. The bacterial community network in the shoots of the E+ plants was more complex than that in the shoots of the E− plants and presented a greater proportion of positive correlations among microorganisms, indicating greater interaction and niche sharing potential among microbes in the E+ plants. These differences may be attributed to *Epichloë* endophyte infection altering the bacterial community structure in the shoot. These findings increase the understanding of the biodiversity–stability relationship and the effect of biodiversity on ecosystem function in microbial ecosystems and suggest that *Epichloë* endophytes play pivotal roles in the assembly and maintenance of host phyllosphere bacterial communities.

### 4.4. Effects of the Epichloë Gansuensis Endophyte Infection on Root Metabolites of Achnatherum Inebrians

Root exudates act as signaling substances, playing a key role in mediating root-root recognition and serving as mediators of plant mineral acquisition under low-nutrient conditions [68]. *Epichloë* endophyte–infected and *Epichloë* endophyte–free plants presented distinct metabolic profiles even under optimal conditions, indicating that *E. gansuensis* endophyte infection is a key driving factor for metabolic profile alterations in *A. inebrians* [25]. Notably, we observed that the *E. gansuensis* induced significant changes in both the quantity and composition of root exudates in *A. inebrians* under low-concentration urea treatment, which aligns with previous findings on low-nitrogen responses [24,29]. The general and specialized metabolic pathways in the roots of the E+ plants were markedly different from those in the roots of the E− plants, reflecting the accumulation of diverse organic acids, amino acids, and fatty acids as reported in previous studies [24,29]. Other metabolomic studies have suggested that endophyte infection modulates host root growth, development, and stress resistance by altering the composition of root exudates and associated metabolic pathways [69,70]. We found that the relative abundances of the plant growth regulator (2-naphthalenyloxy) acetic acid and the alkaloid norajmaline in *Epichloë* endophyte–infected plants were greater than those in *Epichloë* endophyte–free plants. The *Epichloë* endophyte significantly increased the average root diameter and the dry weight of both the shoots and roots, suggesting that the *Epichloë* endophyte may play a key role in plant growth by regulating root metabolism.

### 4.5. Root Microbial and Metabolic Interactions with the Symbiont

Root exudates play specialized roles in mediating biological nutrient cycling, facilitating signal transduction within root–soil–microbiome agroecosystems, and contributing to host responses to both abiotic and biotic stresses [71]. Our previous study demonstrated a significant positive correlation between root metabolite levels and root-associated bacterial taxa under ammonium nitrogen treatment [29]. Consistent with this, a more complex network structure and far more positive interconnections were observed between root exudates and root bacterial communities under urea treatment, indicating the possibility that the *Epichloë* endophyte recruits root microbiota by modulating the metabolites of the host grass. In addition, the co-occurrence network demonstrated that ergometrine, norajmaline, and (2-naphthalenyloxy) acetic acid were positively related to 11 bacterial genera belonging to the Pseudomonadota, Actinobacteriota, Bacteroidota, and Verrucomicrobiota phyla, whereas serylserine and 6-allyl-8b-carboxy-ergoline were negatively associated with these 11 bacterial genera, suggesting a potential beneficial effect of *Epichloë* endophyte infection in enhancing plant adaptation to low-nutrient environments. The alkaloid content of *A. inebrians* increases under salt, drought, and low nitrogen stresses, with a particularly notable increase in the cytotoxic alkaloid ergonovine [72]. The contribution of toxic pyrrolopyrazine and ergot alkaloids to nitrogen uptake and translocation in the shoots of the E+ plants may be linked to the simultaneous accumulation of carbohydrates and amino acids [73]. The strong correlation between specialized root metabolites and the root bacterial community suggests that *Epichloë* endophyte–mediated root exudate profiles may play a role in selectively recruiting beneficial microbiota to colonize the roots of *A. inebrians* in response to urea addition. Overall, advances in amplicon and metabolomic studies have generated information-rich omics datasets revealing the involvement of the bacterial microbial community in the mechanisms of high-efficiency use of nitrogen through interactions with their host at the metabolic level.

## 5. Conclusions

Overall, our research demonstrated the plant growth-promoting ability and differential nitrogen source preferences of the *E. gansuensis* endophyte strain themselves. Meanwhile, it further indicated that the alteration patterns and interaction complexity of the microbiome, and characterization of root metabolism in *A. inebrians* mediated by the *E. gansuensis* endophyte infection under low nitrogen conditions. The *Epichloë* endophyte infection makes a prominent contribution in enhancing the positive interaction between microorganisms and metabolites and promoting plant growth. These findings enhance our understanding of the impact of *Epichloë* endophyte infection on the microbial community composition of host plants and offer new avenues for exploring the complex tripartite interactions among *Epichloë* endophytes, hosts, and microbes.

## Data Availability

16S rRNA amplicon sequencing data in this study are available at the NCBI repository (https://www.ncbi.nlm.nih.gov/) under the accession number SUB13084834.

## Supplementary Materials

The following supporting information can be downloaded at: https://www.mdpi.com/article/10.3390/microorganisms13071493/s1, Figure S1: The growth of *Epichloë gansuensis* strains. (a) colony morphology; (b) iron-carrying capacity testing; Figure S2: The effects of *Epichloë gansuensis* on total C and N in shoots and roots of *Achnatherum inebrians* under low-concentration urea treatment; Figure S3: The bacterial α-diversity in shoot and root of *Achnatherum inebrians*. (a and b) Shannon and (c and d) Chao 1 indexes in shoot and root; Figure S4: Principal Component Analysis (PCA) showing overall metabolic differences between endophyte-infected (E+) and endophyte-free plants (E–) under urea treatment and the magnitude of variability between E+ and E− plants samples within groups; Figure S5: Metabolic pathways involved in root metabolites of endophyte-infected (E+) and endophyte-free (E–) plants; Figure S6: The KEGG enrichment analysis showed significantly different metabolic pathways between E+ and E− plants under urea treatment; Figure S7: Correlation heatmap plots visualizing the relationship between root metabolites and bacterial communities in E+ and E− plants under urea treatment. X-axis: bacterial communities; Y-axis: metabolic compounds; Red and blue boxes: positive or negative correlation; the intensity of the color: correlation R-value; *: correlation significance; *, *p* ≤ 0.05; **, *p* ≤ 0.01; ***, *p* ≤ 0.001. Table S1: Primers and PCR conditions used for PCR amplification of *Epichloë* endophyte strain; Table S2: the overall growth properties of *Epichloë* endophyte strains; Supplemental File S1: Species correlation network of bacteria; Supplemental File S2: Coefficient and significance between node 1 and node 2 in correlation network; Supplemental File S3: Differential Metabolites between E+ and E− plants.
