# Supplementary material for: Achnatherum inebrians Bacterial Communities Associated with Epichloë gansuensis Endophyte Infection Under Low-Concentration Urea Treatment: Links to Plant Growth and Root Metabolite
Source: Microorganisms. 2025 Jun 26;13(7):1493. doi: 10.3390/microorganisms13071493 (PMC12300119; doi:10.3390/microorganisms13071493)
Supplement: Supplementary file 1 [file microorganisms-13-01493-s001.zip › microorganisms-3660050-supplementary.pdf]

# Influence of *Epichloë gansuensis* endophyte on Growth, Root Metabolites, and Microbiome of *Achnatherum inebrians* under Low-Concentration Urea Treatment

**Yuanyuan Jin, Zhenjiang Chen *, Kamran Malik and Chunjie Lia ***

## 2. Materials and methods

### *2.1 Plant material*

*Achnatherum inebrians* seeds from an Tianzhu ecotype whose mature reproductive tillers were originally grown in field environments in Gansu Province, China (Tianzhu, N: 36.97; E: 103.77; H: 2940 m) were used for this experiment. *Epichloë* endophyte infection in seeds and leaf sheaths was examined microscopically via the staining method described by [37]. E- seeds were obtained by killing endophyte viability through treatment with a 100-fold dilution of thiophanate–methyl (Jiangsu Rotam Chemistry Co. Ltd., Jiangsu, China) in 2017. To eliminate potential negative effects of the fungicide on the health of *A. inebrians* plants, the treated seeds were carefully rinsed with water. The E+ and E- plants were cultivated in the field, and seeds were collected annually. Staining microscopic examination and PCR analyses using the following an *Epichloë*–specific PCR primer pair (tub2-exon 1d-1: GAGAAAATGCGTGAGATTGT; tub2-exon 4u-2: GTTTCGTCCGAGTTCTCGAC) [38] was used for screening E- seeds. This ensures that the E- seeds were free of *Epichloë* endophytes. Seeds collected in July 2022, were stored at 4°C to preserve endophyte viability. For the experiment, two hundred E+ and two hundred E- seeds were surface-disinfected with water: sodium hypochlorite (1:1 v/v) and then germinated on seedling-raising plates with sterile vermiculite in a constant-temperature greenhouse (22°C).

### *2.2. Isolation and identification of Epichloë endophyte*

Two months after germination, some E+ seedlings were transferred to the laboratory, washed with distilled water, and cut into small pieces. The E+ seeds and pieces were surface sterilized using 75% ethanol for 30 s and 1.0% NaOCl for 1 min, followed by thorough rinsing with sterilized water 5 times. *Epichloë* endophytes were isolated from sterilized seeds and seedlings of *A. inebrians* placed on potato dextrose agar (PDA), as described by [39]. From 10 to 30 days after incubation, the *Epichloë* endophyte grew and was isolated as a pure culture on fresh PDA medium through three rounds of purification.

*2.3. Epichloë Endophyte Strain Identification*

Fungal DNA was extracted using the Omega D3195-01 HP Fungal DNA Mini Kit following the manufacturer's instructions. Fungal DNA was amplified on a CFX96 optical real-time detection system (CFX96TM Thermal Cycler, USA). The respective reaction mixtures were composed, and the PCR conditions are shown in Table S1. Each qPCR mixture was prepared in a total volume of 25 μL, consisting of 12.5 μL of 2X Taq Master Mix, 9.5 μL of dd$H_2O$, 2.0 μL of forward and reverse primers, and 1 μL of fungal DNA template. Triplicate independent PCRs were conducted for each DNA sample. The gel-purified PCR products were sequenced bidirectionally by Shenggong Bioengineering (Shanghai, China) Co., Ltd. and bidirectionally spliced. BLAST comparisons of the sequences were performed using the NCBI database (https://www.ncbi.nlm.nih.gov/), and the sequences were matched to published ITS for homologous identification.

*2.4. Plant growth-promoting effects of Epichloë Endophyte Strain*

*2.4.1 Production of indole acetic acid (IAA)*

The purified bacterial colonies were picked using a wire loop and added to LB liquid medium containing L-tryptophan (10 g tryptone, 5 g yeast, 10 g NaCl, 1 L distilled water, 5 mmol/L L-tryptophan, pH 7.2, sterilization at 121℃ for 30 min, cooled to room temperature). The medium without bacterial addition (control group) was placed in incubation at 28℃ with 120 r.min$^{-1}$ for 5 days. The fungal solution (1 mL) was taken into a 1.5 mL sterilized centrifuge tube, and centrifuged at 12000 rpm for 5 min. Color development solution (200 μL) was separately added to aliquots of the supernatant and indoleacetic acid standard solution and placed for 30 min at the room temperature under the light proof condition to observe the color change of reaction mix. No potential for growth hormone secretion if color remains unchanged. A 200 μL of the color development reaction mixture was immediately withdrawn and added to a 96-well plate, and the value was read at OD530 using an enzyme marker. The standard curve of IAA concentration was plotted on the basis of the value of indoleacetic acid standard solution read as the vertical coordinate and its concentration as the horizontal coordinate, and then the concentration of IAA in the fungal solution was calculated on the basis of the value at OD530.

2.4.2 Iron carrier detection

Isolated single colonies were inoculated into sterilized MKB medium (15 mL glycerin, 3.28 g $FeCl_3 \cdot 6H_2O$, 5 g acid hydrolyzed casein, 2.5 g $MgSO_4 \cdot 7H_2O$ and 18g AGAR powder) by using the inoculation loop, and incubated for 2 days at 28°C in inverted position. After growing distinct single colonies, 10 ml of CAS solution cooled at 60°C was added to each inoculated MKB plate.

MKB medium without fungal inoculation was used as a blank control. The plates were left for 1h to observe the color change of each plate.

2.4.3 Detection of nitrogen fixation potential

The isolated strains were inoculated onto Ashby's medium (10 g glucose, 0.2 g $KH_2PO_4$, 0.2 g $MgSO_4 \cdot 7H_2O$, 0.2 g NaCl, 0.1 g $CaSO_4 \cdot 2H_2O$, 5 g $CaCO_3$, 15 g agar, 1000 mL distilled water and pH 7.0~7.2) and incubated in inverted culture at 28°C for 4-7 days. Fungi with nitrogen fixation would grow colonies on nitrogen-free media. Meanwhile, extracted fungal DNA was amplified for the nitrogen fixation gene *nifH*. The amplified PCR products were detected by 2% agarose gel electrophoresis.

*2.5. Experiment 1: Effects of Nitrogen Fertilizers on Epichloë Endophyte Strains*

To study the effects of different nitrogen sources ($NH_4^+$-N, $NO_3^-$-N, and urea-N) on the growth of the *Epichloë* endophyte strain, basal culture media were prepared using 200 g of peeled potato (*Solanum tuberosum*), 20 g of glucose, 18 g of agar (liquid medium without agar) and 1000 mL water. Urea (10.72 g), $NH_4Cl$ (11.12 g) and $NaNO_3$ (17.86 g) were added to the basal medium to create media with different nitrogen sources, and N content was 0.15 mol/L, 0.1 mol/L and 0.1 mol/L. Solid and liquid PDA medium without N was used as a control. The solid medium was dispensed into 90 mm diameter Petri dishes, with approximately 30 mL per dish. After the Petri dishes had cooled, a layer of breathable film (cellophane) was placed on top of the medium in each dish. The liquid medium was transferred to 200 mL triangular flask, with approximately 50 mL per flask. The experiment was conducted with five replicates for each nitrogen fertilizer treatment. The *Epichloë* endophyte strain was tested for its ability to utilize organic and inorganic nitrogen sources in both solid and liquid media. The *Epichloë* endophyte strain was incubated in liquid medium at 25°C for 10 days with shaking at 150 x g. The colony diameter of the fungal endophytes on solid media was measured using Vernier calipers, whereas the mycelial concentration of the fungal endophytes in liquid media was measured using the drying and weighing method.

*2.6. Experiment 2: Effects of Urea on the Achnatherum Inebrians–Epichloë Endophyte Symbiont*

*2.6.1. Experimental Design*

On the basis of the results of experiment 1, urea was selected as the nitrogen source to study the effects of low-nitrogen treatment on the growth, bacterial microorganisms and root metabolites of the *A. inebrians–Epichloë* endophyte symbiont. Our previous study revealed that *Epichloë* endophyte infection significantly promoted the nitrogen utilization use efficiency of host plants under 0.1 mmol/L low-nitrogen treatment [29, 44]. Therefore, we chose 0.1 mmol/L as the stress concentration for urea. After germination for 2 months, 10 cm-tall seedlings were transplanted into hydroponic boxes measuring 40 cm in length, 26 cm in width, and 25 cm in height. Each box, which contained six seedlings, was filled with 400 mL of modified ½-strength

Hoagland's nutrient solution (urea as a nitrogen source). A pot experiment with a completely randomized design with a factorial arrangement of treatments was established in an artificial greenhouse. The treatments included a single genotype of *A. inebrians* plants with two levels of endophyte infection (E+ and E-) and one nitrogen level. Each treatment was replicated four times. During the 63-day growth period, E+ and E- plants were cultivated at average day and night temperatures of 25±2°C and 18±2°C, respectively, with a 16 h: 8 h, light: dark photoperiod (c. 600 μmol $m^{-2}$. $s^{-1}$). Plant height and root length were measured using a ruler before harvest. Leaf and root tissues from E+ and E- plants (n = 6) subjected to low-nitrogen treatment were collected after 63 days of growth. 6 plants were analyzed per treatment group, totaling 72 plants. The roots of E+ and E- plants were washed with deionized water and transferred to aluminum foil-wrapped conical flasks filled with low-nitrogen nutrient solution [45]. E+ and E- plants were incubated under a normal photoperiod (16 h light/8 h dark) at 26°C for 7 d, after which the culture liquid was collected for metabolite profiling by filtration through a 0.22 μm membrane. The total length, diameter, volume, surface area, tip and fork number of the roots were analyzed using a Perfection V550 scanner (Epson America Inc).

*2.6.2. Nutrient Content and Dry Weight Measurements of Shoots and Roots*

The total carbon content in the shoots and roots of the E+ and E- plants was determined by using a CHNS/O analyzer (Flash EA 1112 Series, Italy). Shoots and roots from the E+ and E- samples were digested with $H_2SO_4$ solution and a catalyst ($CuSO_4$:$K_2SO_4$: 1:10 mixture) at 420°C for 1 h. The concentrations of total nitrogen (N) and total phosphorus (P) were then determined using an injection system (FIAstar 5000 Analyzer, Foss, Denmark). A constant weight was achieved by oven drying the samples at 80°C for 48 h, and the dry weight was determined with an electronic balance.

*2.6.3. DNA Extraction and Sequencing*

Total DNA extraction from the shoots and roots of E+ and E- plants (n=6) was carried out using a modified CTAB DNA extraction method [46]. The concentration and purity of DNA were measured using a Micro NanoDrop ND-1000 UV–Vis Spectrophotometer (Nanodrop Technologies, Wilmington, DE, USA). The quality of DNA was evaluated via 1% agarose gel electrophoresis and then was stored at -20°C.

10-ng DNA per sample was used to amplify the V3-V4 region of the 16S rRNA gene using an ABI GeneAmp®9700 PCR systems. The amplification was performed using Trans Start FastPfu DNA Polymerase (TransGen AP221-02) and specific primer (338F: 5'-ACTCCTACGGGAGGCAGCAG-3' and 806R: 5'-GGACTACHVGGGTWTCTAAT-3') [47]. The PCR products from the same samples were pooled and analyzed via 2% agarose gel electrophoresis. The PCR products were recovered from the gel using the AxyPrepDNA Gel Recovery Kit (AXYGEN Co.) and eluted with Tris-HCl. Four biological replicates with the highest DNA quality were screened for *16S rRNA* amplicon sequencing, which was performed on an

Illumina MiSeq PE300 (Illumina Inc., San Diego, CA, USA) sequencing platform by Shanghai Majorbio Bio-Pharm Technology Co. [43].

*2.6.4. Metabolite Extraction and UHPLC–MS/MS Analysis*

Metabolite analysis was conducted following previously established protocols [48]. 400 µL of a methanol: acetonitrile (1:1, v/v) mixture was added to 100 µL of the root extraction mixture collected from the E+ and E- plants, respectively. The mixture was sonicated at 40 kHz for 30 min at 5°C. Samples were incubated at -20°C for 30 min to precipitate the proteins, followed by centrifugation at 13,000 x g for 15 min at 4°C. The resulting supernatants were transferred to fresh vials and dried under a gentle stream of nitrogen gas. After drying the metabolites were reconstituted in 100 µL of an acetonitrile: water (1:1, v/v) mixture by brief sonication in a water bath maintained at 5°C. The reconstituted samples were then centrifuged at 13,000 x g for 15 min at 4°C. The supernatants stored at -80°C until ultrahigh-performance liquid chromatography–tandem mass spectrometry (UHPLC–MS/MS) analysis.

To investigate root metabolic N responses in the E+ plants, UHPLC–MS/MS analysis was used to determine the main root exudates in the E+ and E- plants. Chromatographic separation of the metabolites was performed using a UHPLC system coupled with an electrospray ionization (ESI) source operating in either positive (POS) or negative (NEG) ion mode. The analysis followed a previously established protocol [49]. Briefly, metabolite separation was performed by injecting a 2 µL sample onto a Waters HSS T3 column (100 mm × 2.1 mm, 1.8 µm). The mobile phase consisted of solvent A (water with 0.1% formic acid: acetonitrile, 95:5, v/v) and solvent B (acetonitrile: isopropanol: water with 0.1% formic acid, 47.5: 47.5: 5, v/v). The separation was carried out at a flow rate of 0.4 mL/min. Quality control (QC) samples and reference samples were injected every ten samples during the analytical run to monitor and evaluate the stability of the LC–MS system.

*2.6.5. LC–MS Data Processing and Annotation*

Following UHPLC–MS/MS analyses, the data acquired through data-dependent MS/MS acquisition (DDA) were processed using Progenesis QI 3.0 (Nonlinear Dynamics, Waters, USA) for data preprocessing. The processing steps included baseline filtering, retention time (RT) alignment, lock-in mass correction, peak recognition and integration (excluding low-quality peaks), adduct grouping, and deconvolution. The parameters used were as follows: peak picking with the automatic sensitivity method (default settings) and an RT range of 0.1-8.0 min. Annotated metabolites were identified on the basis of retention time (RT) and m/z information (designated as features). Prior to downstream statistical analyses, ion abundances were normalized to the internal standard telmisartan by sum, on the basis of four biological replicates. Subsequent multivariate analyses, including Venn analysis principal component analysis (PCA) and partial least squares discriminant analysis (PLS–DA), were conducted using the Venn Diagram and ropls package (v1.20.0) in R [50]. Feature annotations were conducted using the Kyoto Encyclopedia of Genes and Genomes (KEGG, http://www.genome.jp/kegg/), Metlin

(https://metlin.scripps.edu/), Human Metabolome (HMDB, http://www.hmdb.ca/) and Majorbio databases based on 10 ppm precursor mass tolerance, 95% isotope similarity and 10 ppm fragment mass tolerance. To accurately evaluate the metabolic features of the root exudates between endophyte-infected plants and endophyte-free plants under urea treatment, 564 positive mode mass features (identified as RT: m/z ratio pairs) after data preprocessing were used for downstream statistical analysis.

*2.6.6. Mining of Differentially Abundant Metabolites*

Multivariate statistical analysis was performed on the Majorbio Cloud Platform (https://cloud.majorbio.com). Orthogonal least partial squares discriminant analysis (OPLS–DA) was performed using the ROPLS package (v 1.6.2) in R to identify metabolic changes between the E+ and E- samples. The stability and reliability of the model were evaluated using 7-fold cross-validation and response permutation testing. Differentially expressed metabolites were identified on basis of the variable importance in the projection (VIP) score and the following parameters: VIP>1; *P* value of Student's t test <0.05; and fold change (FC) <1 or FC>1, using the default settings. The biochemical pathways of the significantly different metabolites were mapped with metabolic enrichment and pathway analysis using the KEGG database. Significantly enriched pathways were identified using Fisher's exact test ($P < 0.05$) implemented in the Stats 2.0 package in R and the SciPy package (v 1.11.4) (https://docs.scipy.org/doc/scipy/).

*2.7. Bioinformatics Analysis*

The raw sequence reads obtained via Illumina sequencing were first assembled based on overlap relationships and then quality-filtered and trimmed using fastp v0.19.6 with adapter autodetection and the default parameters [51]. Low-quality reads (length < 50 bp or with a quality value < 20 or having N bases were removed. The quality-filtered reads (34000-43000 sequences) were processed using the QIIME 2.0 pipeline on the free online platform of Majorbio Cloud (www.majorbio.com). The optimized reads were further processed using sequence noise reduction methods (DADA2) to generate representative amplicon sequence variant (ASV) sequences and their corresponding abundance information. To determine the species classification for each ASV, the Silva database on the QIIME (from Latin *Silva*, forest, http://www.arb-silva.de) on Qiime 2.0 platform (https://qiime2.org) was used to analyze the taxonomy of the ASV representative sequences.

The number of ASVs shared and unique between the shoots and roots of the E+ and E- plants was analyzed using a Venn diagram generated by VennDiagram package (v1.7.3) in R (v. 3.3.1). Relative abundances at the bacterial class taxonomic rank were analyzed and visualized for the 30 most abundant phyla using bar a chart. The Wilcoxon rank-sum test was used to detect species differing in abundance in the microbial community between the E+ and E- plants under urea treatment. The Shannon and Chao1 diversity indices were calculated via Student's t test by Mothur v1.30.2 (https://mothur.org/wiki/download_mothur/) software in R. Principal coordinate analysis (PCoA) was performed with the ordinate phyloseq R function by first transforming

relative abundances into a Bray–Curtis dissimilarity matrix using the vegdist function and differences in species composition between the E+ and E- samples were calculated using the Adonis function within the vegan v3.3.1 R package.

## 2.8. Statistical Analyses

The statistically significant differences in plant biomass, plant nutrients and bacterial community diversity between E+ and E- plants were evaluated by an independent sample t-test at a threshold P-value < 0.05. A significant effects of different N sources on colony diameter and mycelial concentration of E. gansuensis endophyte strain was analyzed with a one-way ANOVA ($P < 0.05$). All analyses of variance (ANOVA) were performed using SPSS statistical software (Version 20.0, Inc., Chicago, IL).

## 2.9. Analysis of the Microbial Co- occurrence Networks in the Shoot, Root, and Root Microbiota–Metabolite Interactions

We elucidated the co-occurrence correlations between metabolomics and microbiomics using network analysis under U-N treatment, focusing on differential root metabolites and root bacterial microbes at the genus level on the basis of Spearman's rank correlation coefficient. Spearman's rank correlation coefficient was calculated among microorganisms and between microbes and metabolites. The obtained numerical matrix was used for a hierarchical clustering with complete linkages and visualized using the heatmap function within the Heatmap v3.3.1 R package. To investigate how microbial interactions differ between the shoots and roots of *A. inebrians*, microbial co-occurrence networks were constructed using the "NETWORKX v1.11" python package on basis of significant pairwise Spearman's correlations between bacterial microbial taxa in the shoots and roots of the E+ and E- plants [52]. The network complexity was then compared between the shoots and roots as well as between the E+ and E- plants. The nodes and edges in the network represent bacterial ASVs and significant interactions between pairs of ASVs; for a cumulative degree distribution see SUPPLEMENTAL FILES 1 and 2. To identify metabolites that play a key role, we investigated the correlation between root bacteria and root exudates using a two-factor correlation network analysis. The ASVs with relative abundances of less than 0.01% in the E+ and E- samples were filtered out because they were poorly represented [53]. Only rank correlation coefficients with values greater than 0.7 or less than -0.7 and a statistically significant *P* value ($P< 0.05$) were considered valid correlations in the network. The bacterial networks in the shoots and roots were graphically visualized using Cytoscape. To assess the response of microbial interactions in the shoot and root, as well as root microbial–metabolite interactions under urea nitrogen supply, six network properties were quantified: network diameter, degree, closeness centrality, betweenness centrality, degree centrality, and degree distribution. These metrics have been commonly used in previous studies to evaluate microbial community stability [54].

**Table S1 Primers and PCR conditions used for PCR amplification of *Epichloë* endophyte strain.**

| Target gene | Primers | Sequence(5'-3') | Thermal profile | Number of cycles | Reference |
|---|---|---|---|---|---|
| ITS | ITS1 | TCCGTAGGTGAACCTGCGC | 94°C, 3 min; 94°C, 30 s; 55°C, 30 s; 72°C, 50 s; 72°C, 10 min. | 30 | (White et al., 1990) |
| | ITS4 | TCCTCCGCTTATTGATATGC | | | |

**Table S2 the overall growth properties of *Epichloë* endophyte strains.**

| Name | NCBI Comparison | Ferritin carrier | Indole acetic acid | Pectinase | nitrogen-free agar media |
|---|---|---|---|---|---|
| *Epichloë gansμensis* | 97.82% | without | without | without | failure to grow |

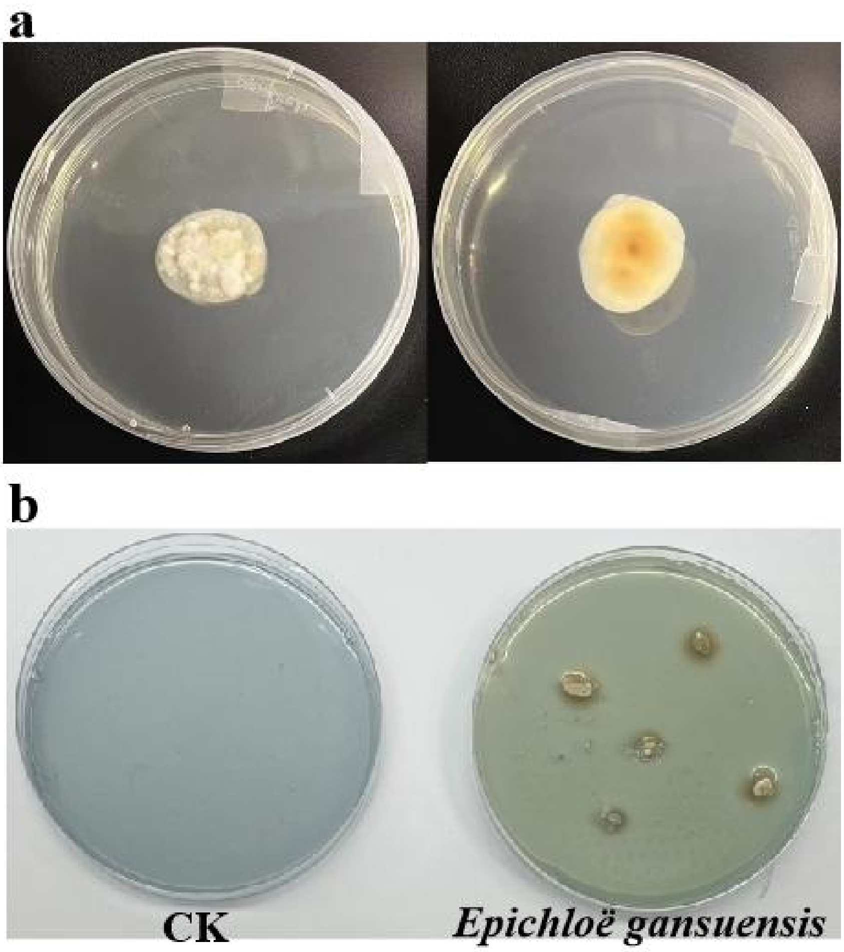


**Figure S1** The growth of *Epichloë gansuensis* strains. (a) colony morphology; (b) iron-carrying capacity testing.

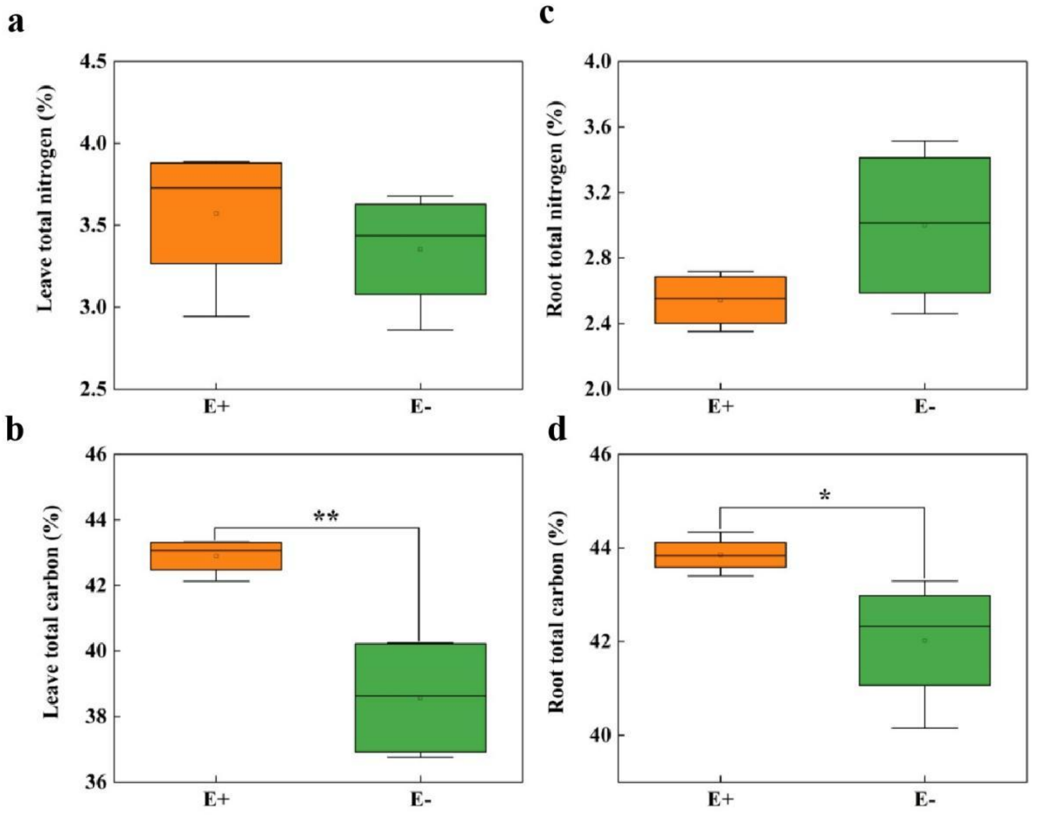


**Figure S2** The effects of *Epichloë gansuensis* on total C and N in shoots and roots of *Achnatherum inebrians* under low-concentration urea treatment.

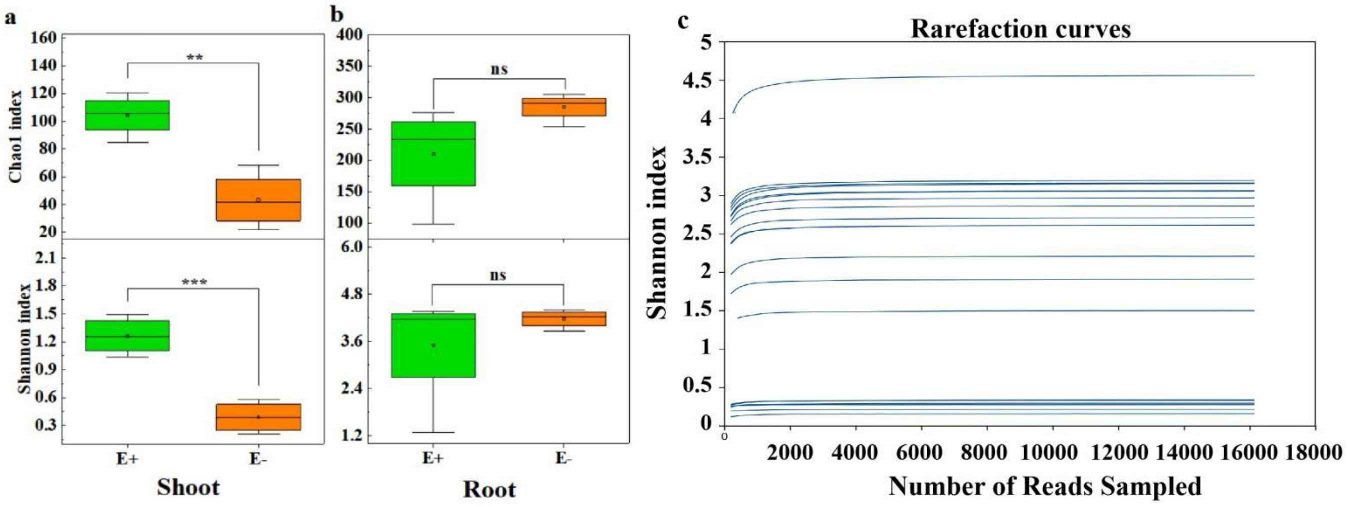


**Figure S3** The bacterial α-diversity in shoot and root of *Achnatherum inebrians.* (a and b) Shannon and (c and d) Chao 1 indexes in shoot and root.

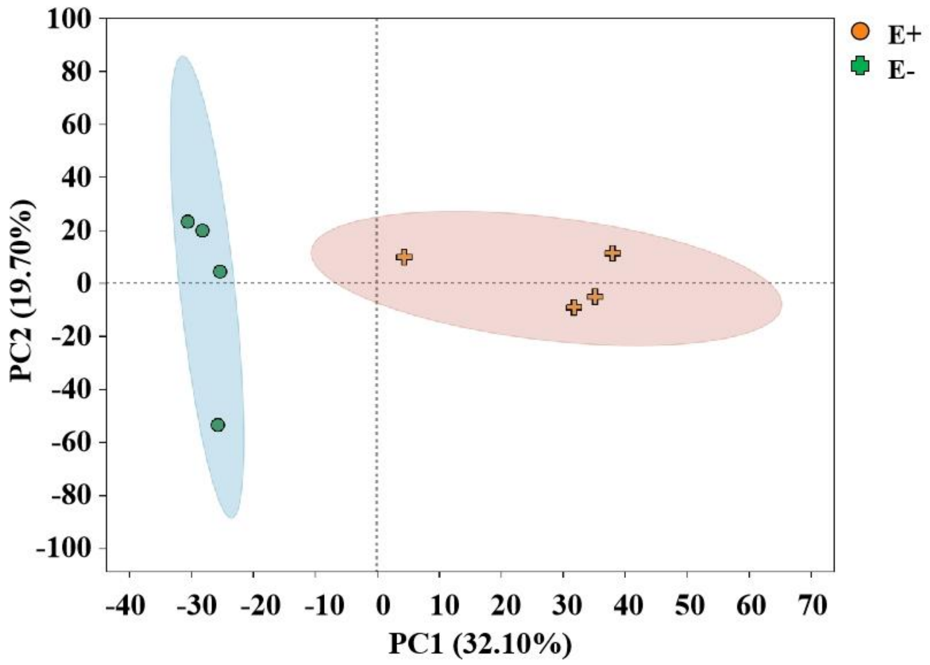


**Figure S4** Principal Component Analysis (PCA) showing overall metabolic differences between endophyte-infected (E+) and endophyte-free plants (E-) under urea treatment and the magnitude of variability between E+ and E- plants samples within groups.

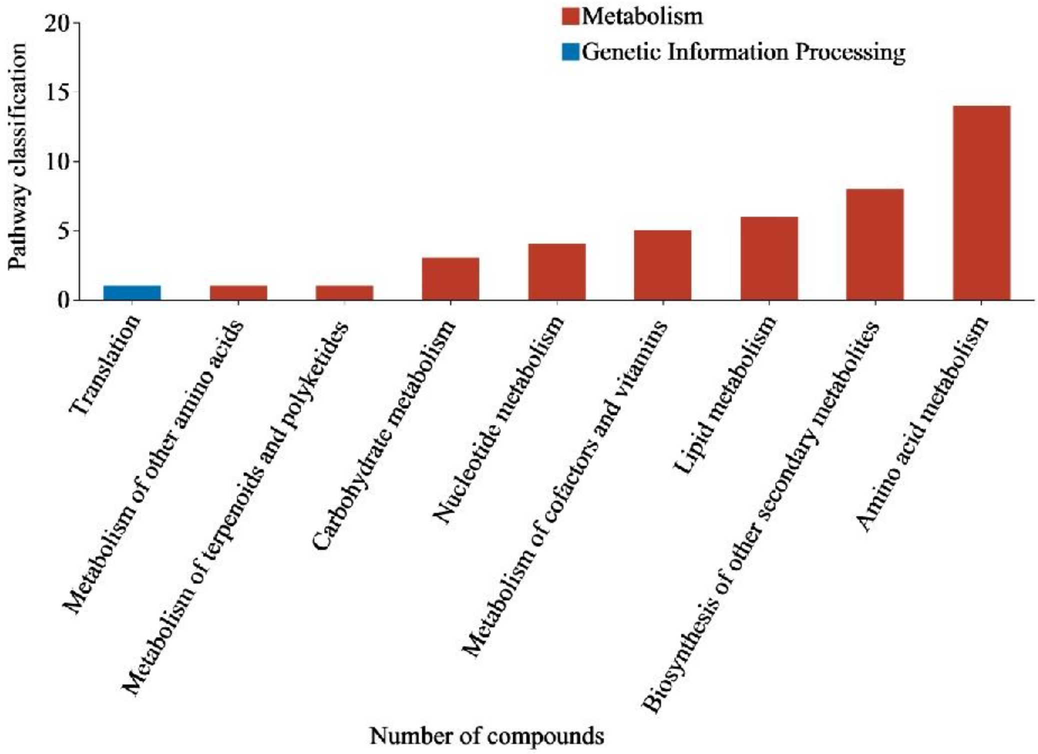


**Figure S5** Metabolic pathways involved in root metabolites of endophyte-infected (E+) and endophyte-free (E-) plants.

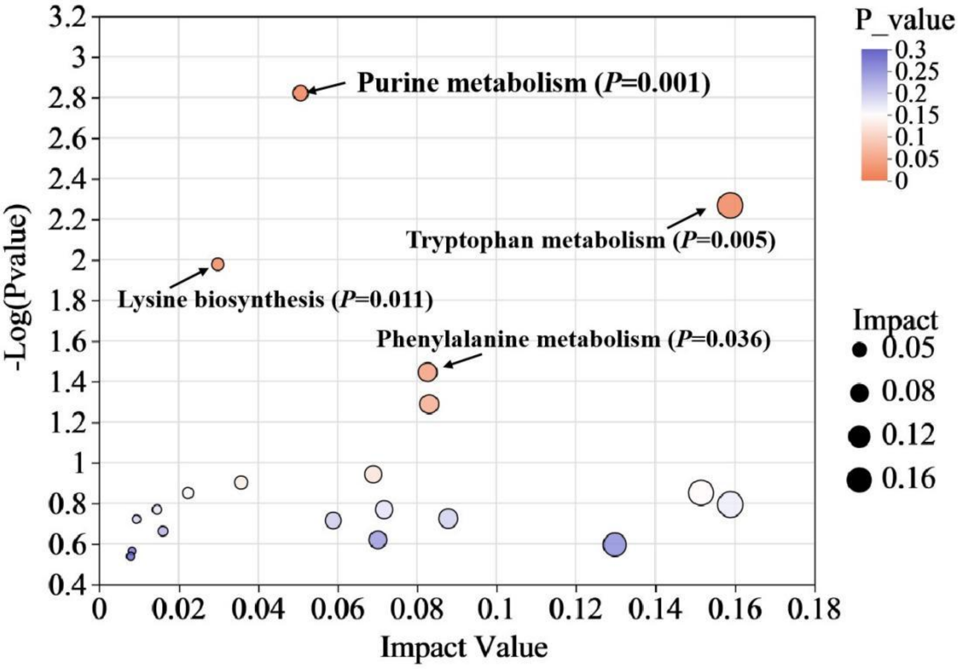


**Figure S6** The KEGG enrichment analysis showed significantly different metabolic pathways between E+ and E- plants under urea treatment.

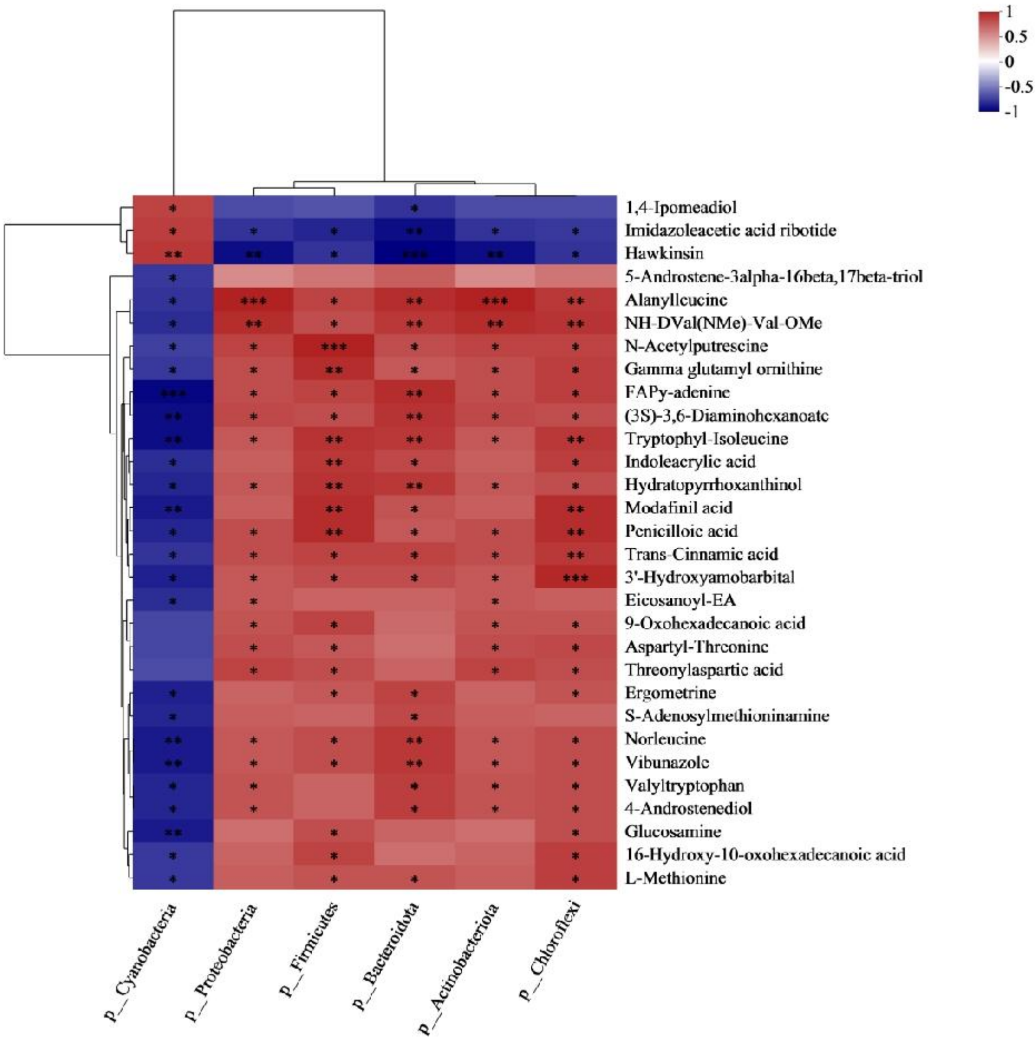


**Figure S7** Correlation heatmap plots visualizing the relationship between root metabolites and bacterial communities in E+ and E- plants under urea treatment. X-axis: bacterial communities; Y-axis: metabolic compounds; Red and blue boxes: positive or negative correlation; the intensity of the color: correlation R-value; *: correlation significance; *, $P \leq 0.05$; **, $P \leq 0.01$; ***, $P \leq 0.001$.

**SUPPLEMENTAL FILE S1**

**Table 1** species correlation network of bacteria in shoot of E+ and E- plants.

| Node_ID | Node_Name | Degree | Clustering | Degree_Centrality | Closeness_Centrality | Betweenness_Centrality |
|---|---|---|---|---|---|---|
| 1 | *g__Acidovorax* | 2 | 0 | 0.045454545 | 0.081818182 | 0.005285412 |
| 2 | *g__Acinetobacter* | 2 | 0 | 0.045454545 | 0.11654334 | 0.021141649 |
| 3 | *g__Aeromicrobium* | 5 | 1 | 0.113636364 | 0.141165173 | 0 |
| 4 | *g__Allorhizobium-Neorhizobium-Pararhizobium-Rhizobium* | 2 | 1 | 0.045454545 | 0.05861244 | 0 |
| 5 | *g__Aquabacterium* | 8 | 0.5357 | 0.181818182 | 0.182231405 | 0.0602537 |
| 6 | *g__Azospirillum* | 3 | 0 | 0.068181818 | 0.178977273 | 0.122621564 |
| 7 | *g__Bauldia* | 3 | 1 | 0.068181818 | 0.068181818 | 0 |
| 8 | *g__Bdellovibrio* | 4 | 1 | 0.090909091 | 0.090909091 | 0 |
| 9 | *g__Bosea* | 3 | 0.3333 | 0.068181818 | 0.079545455 | 0.010570825 |
| 10 | *g__Brachybacterium* | 3 | 1 | 0.068181818 | 0.068181818 | 0 |
| 11 | *g__Brevundimonas* | 4 | 1 | 0.090909091 | 0.090909091 | 0 |
| 12 | *g__Cellvibrio* | 4 | 1 | 0.090909091 | 0.111363636 | 0 |
| 13 | *g__Chryseobacterium* | 6 | 0.4667 | 0.136363636 | 0.137297634 | 0.071881607 |
| 14 | *g__Delftia* | 4 | 0.1667 | 0.090909091 | 0.161656891 | 0.109936575 |
| 15 | *g__Devosia* | 1 | 0 | 0.022727273 | 0.113894628 | 0 |
| 16 | *g__Enterobacter* | 5 | 1 | 0.113636364 | 0.141165173 | 0 |
| 17 | *g__Exiguobacterium* | 1 | 0 | 0.022727273 | 0.094554031 | 0 |
| 18 | *g__Flavihumibacter* | 4 | 1 | 0.090909091 | 0.111363636 | 0 |
| 19 | *g__Flavobacterium* | 1 | 0 | 0.022727273 | 0.122228381 | 0 |
| 20 | *g__Gemmobacter* | 8 | 0.5357 | 0.181818182 | 0.182231405 | 0.0602537 |

| | | | | | | |
|---|---|---|---|---|---|---|
| 21 | *g__Herpetosiphon* | 1 | 0 | 0.022727273 | 0.022727273 | 0 |
| 22 | *g__Hydrogenophaga* | 5 | 0.6 | 0.113636364 | 0.116883117 | 0.00845666 |
| 23 | *g__Hyphomonas* | 3 | 1 | 0.068181818 | 0.068181818 | 0 |
| 24 | *g__Jeotgalibacillus* | 1 | 0 | 0.022727273 | 0.13187799 | 0 |
| 25 | *g__Microbacterium* | 1 | 0 | 0.022727273 | 0.054545455 | 0 |
| 26 | *g__Mycobacterium* | 4 | 1 | 0.090909091 | 0.111363636 | 0 |
| 27 | *g__norank_f__67-14* | 3 | 1 | 0.068181818 | 0.069602273 | 0 |
| 28 | *g__norank_f__Solirubrobacteraceae* | 4 | 1 | 0.090909091 | 0.090909091 | 0 |
| 29 | *g__norank_o__Microtrichales* | 3 | 1 | 0.068181818 | 0.068181818 | 0 |
| 30 | *g__norank_p__WPS-2* | 3 | 1 | 0.068181818 | 0.069602273 | 0 |
| 31 | *g__Novosphingobium* | 2 | 0 | 0.045454545 | 0.09280303 | 0.012684989 |
| 32 | *g__Paenarthrobacter* | 5 | 0.5 | 0.113636364 | 0.147393048 | 0.022198732 |
| 33 | *g__Paracoccus* | 4 | 0.5 | 0.090909091 | 0.147393048 | 0.040169133 |
| 34 | *g__Planococcus* | 6 | 0.8 | 0.136363636 | 0.145256917 | 0.002114165 |
| 35 | *g__Porphyrobacter* | 4 | 0.5 | 0.090909091 | 0.09280303 | 0.012684989 |
| 36 | *g__Pseudomonas* | 2 | 1 | 0.045454545 | 0.05861244 | 0 |
| 37 | *g__Pseudoxanthomonas* | 1 | 0 | 0.022727273 | 0.022727273 | 0 |
| 38 | *g__Reyranella* | 3 | 1 | 0.068181818 | 0.069602273 | 0 |
| 39 | *g__Rhodococcus* | 5 | 1 | 0.113636364 | 0.141165173 | 0 |
| 40 | *g__Sphingobium* | 1 | 0 | 0.022727273 | 0.022727273 | 0 |
| 41 | *g__unclassified_f__Rhizobiaceae* | 2 | 1 | 0.045454545 | 0.130165289 | 0 |
| 42 | *g__unclassified_f__Rhodobacteraceae* | 3 | 0.3333 | 0.068181818 | 0.185606061 | 0.11627907 |
| 43 | *g__unclassified_f__Sphingomonadaceae* | 1 | 0 | 0.022727273 | 0.022727273 | 0 |
| 44 | *g__unclassified_o__Rhizobiales* | 4 | 1 | 0.090909091 | 0.090909091 | 0 |
| 45 | *g__Verrucomicrobium* | 4 | 1 | 0.090909091 | 0.111363636 | 0 |

**Table 2** species correlation network of bacteria in shoot of E+ plants.

| Node_ID | node_name | Degree | Clustering | Degree_Centrality | Closeness_Centrality | Betweenness_Centrality |
|---|---|---|---|---|---|---|
| 1 | *g__Acinetobacter* | 1 | 0 | 0.033333333 | 0.033333333 | 0 |
| 2 | *g__Actinoplanes* | 1 | 0 | 0.033333333 | 0.033333333 | 0 |
| 3 | *g__Aeromicrobium* | 1 | 0 | 0.033333333 | 0.033333333 | 0 |
| 4 | *g__Aquabacterium* | 3 | 1 | 0.1 | 0.1 | 0 |
| 5 | *g__Azospirillum* | 4 | 1 | 0.133333333 | 0.133333333 | 0 |
| 6 | *g__Bdellovibrio* | 6 | 1 | 0.2 | 0.2 | 0 |
| 7 | *g__Brevundimonas* | 6 | 1 | 0.2 | 0.2 | 0 |
| 8 | *g__Cellvibrio* | 4 | 1 | 0.133333333 | 0.133333333 | 0 |
| 9 | *g__Dyadobacter* | 4 | 1 | 0.133333333 | 0.133333333 | 0 |
| 10 | *g__Enterobacter* | 1 | 0 | 0.033333333 | 0.033333333 | 0 |
| 11 | *g__Flavihumibacter* | 4 | 1 | 0.133333333 | 0.133333333 | 0 |
| 12 | *g__Gemmobacter* | 3 | 1 | 0.1 | 0.1 | 0 |
| 13 | *g__Mycobacterium* | 4 | 1 | 0.133333333 | 0.133333333 | 0 |
| 14 | *g__norank_c__Sericytochromatia* | 4 | 1 | 0.133333333 | 0.133333333 | 0 |
| 15 | *g__norank_f__Solirubrobacteraceae* | 6 | 1 | 0.2 | 0.2 | 0 |
| 16 | *g__Novosphingobium* | 3 | 1 | 0.1 | 0.1 | 0 |
| 17 | *g__Paenarthrobacter* | 1 | 0 | 0.033333333 | 0.033333333 | 0 |
| 18 | *g__Paracoccus* | 1 | 0 | 0.033333333 | 0.033333333 | 0 |
| 19 | *g__Porphyrobacter* | 6 | 1 | 0.2 | 0.2 | 0 |
| 20 | *g__Pseudomonas* | 1 | 0 | 0.033333333 | 0.033333333 | 0 |
| 21 | *g__Pseudoxanthomonas* | 6 | 1 | 0.2 | 0.2 | 0 |
| 22 | *g__Rhodobacter* | 4 | 1 | 0.133333333 | 0.133333333 | 0 |
| 23 | *g__Rhodococcus* | 1 | 0 | 0.033333333 | 0.033333333 | 0 |

| | | | | | | |
|---|---|---|---|---|---|---|
| 24 | *g__Sphingobium* | 1 | 0 | 0.033333333 | 0.033333333 | 0 |
| 25 | *g__Sphingomonas* | 6 | 1 | 0.2 | 0.2 | 0 |
| 26 | *g__unclassified_f__Flavobacteriaceae* | 4 | 1 | 0.133333333 | 0.133333333 | 0 |
| 27 | *g__unclassified_f__Microbacteriaceae* | 4 | 1 | 0.133333333 | 0.133333333 | 0 |
| 28 | *g__unclassified_f__Rhizobiaceae* | 3 | 1 | 0.1 | 0.1 | 0 |
| 29 | *g__unclassified_f__Sphingomonadaceae* | 1 | 0 | 0.033333333 | 0.033333333 | 0 |
| 30 | *g__unclassified_o__Rhizobiales* | 6 | 1 | 0.2 | 0.2 | 0 |
| 31 | *g__Verrucomicrobium* | 4 | 1 | 0.133333333 | 0.133333333 | 0 |

**Table 3** species correlation network of bacteria in shoot of E- plants.

| Node_ID | node_name | Degree | Clustering | Degree_Centrality | Closeness_Centrality | Betweenness_Centrality |
|---|---|---|---|---|---|---|
| 1 | *g__Actinoplanes* | 2 | 1 | 0.1 | 0.1 | 0 |
| 2 | *g__Allorhizobium-Neorhizobium-Pararhizobium-Rhizobium* | 1 | 0 | 0.05 | 0.05 | 0 |
| 3 | *g__Bauldia* | 4 | 1 | 0.2 | 0.2 | 0 |
| 4 | *g__Brachybacterium* | 4 | 1 | 0.2 | 0.2 | 0 |
| 5 | *g__Exiguobacterium* | 2 | 1 | 0.1 | 0.1 | 0 |
| 6 | *g__Flavobacterium* | 1 | 0 | 0.05 | 0.05 | 0 |
| 7 | *g__Herpetosiphon* | 2 | 1 | 0.1 | 0.1 | 0 |
| 8 | *g__Hyphomonas* | 4 | 1 | 0.2 | 0.2 | 0 |
| 9 | *g__Jeotgalibacillus* | 1 | 0 | 0.05 | 0.05 | 0 |
| 10 | *g__norank_f__67-14* | 5 | 1 | 0.25 | 0.25 | 0 |
| 11 | *g__norank_o__Microtrichales* | 4 | 1 | 0.2 | 0.2 | 0 |
| 12 | *g__norank_p__WPS-2* | 5 | 1 | 0.25 | 0.25 | 0 |

| | | | | | | |
|---|---|---|---|---|---|---|
| 13 | g__*Novosphingobium* | 5 | 1 | 0.25 | 0.25 | 0 |
| 14 | g__*Paenarthrobacter* | 4 | 1 | 0.2 | 0.2 | 0 |
| 15 | g__*Paracoccus* | 2 | 1 | 0.1 | 0.1 | 0 |
| 16 | g__*Porphyrobacter* | 5 | 1 | 0.25 | 0.25 | 0 |
| 17 | g__*Pseudomonas* | 2 | 1 | 0.1 | 0.1 | 0 |
| 18 | g__*Pseudoxanthomonas* | 2 | 1 | 0.1 | 0.1 | 0 |
| 19 | g__*Reyranella* | 5 | 1 | 0.25 | 0.25 | 0 |
| 20 | g__*Rhodobacter* | 1 | 0 | 0.05 | 0.05 | 0 |
| 21 | g__*unclassified_f__Microbacteriaceae* | 5 | 1 | 0.25 | 0.25 | 0 |

**Table 4** species correlation network of bacteria in root of E+ and E- plants.

| Node_ID | Node_Name | Degree | Clustering | Degree_Centrality | Closeness_Centrality | Betweenness_Centrality |
|---|---|---|---|---|---|---|
| 1 | g__*Acidibacter* | 6 | 0.2 | 0.133333333 | 0.32979066 | 0.201772487 |
| 2 | g__*Acidovorax* | 3 | 1 | 0.066666667 | 0.167320261 | 0 |
| 3 | g__*Acinetobacter* | 3 | 1 | 0.066666667 | 0.175042735 | 0 |
| 4 | g__*Allorhizobium-Neorhizobium-Pararhizobium-Rhizobium* | 1 | 0 | 0.022222222 | 0.033333333 | 0 |
| 5 | g__*Azospirillum* | 1 | 0 | 0.022222222 | 0.16489533 | 0 |
| 6 | g__*Bdellovibrio* | 1 | 0 | 0.022222222 | 0.061538462 | 0 |
| 7 | g__*Bosea* | 3 | 0 | 0.066666667 | 0.237037037 | 0.050925926 |
| 8 | g__*Cellvibrio* | 2 | 1 | 0.044444444 | 0.061538462 | 0 |
| 9 | g__*Chryseobacterium* | 1 | 0 | 0.022222222 | 0.022222222 | 0 |
| 10 | g__*Comamonas* | 2 | 0 | 0.044444444 | 0.212668744 | 0.020875421 |
| 11 | g__*Cytophaga* | 1 | 0 | 0.022222222 | 0.201376598 | 0 |

| | | | | | | |
|---|---|---|---|---|---|---|
| 12 | *g__Devosia* | 8 | 0.3214 | 0.177777778 | 0.27416332 | 0.093350168 |
| 13 | *g__Enterobacter* | 5 | 0.7 | 0.111111111 | 0.242080378 | 0.009873337 |
| 14 | *g__Exiguobacterium* | 2 | 0 | 0.044444444 | 0.05 | 0.002020202 |
| 15 | *g__Flavihumibacter* | 3 | 0.6667 | 0.066666667 | 0.218803419 | 0.007704024 |
| 16 | *g__Flavobacterium* | 3 | 0.3333 | 0.066666667 | 0.088888889 | 0.008080808 |
| 17 | *g__Herpetosiphon* | 3 | 1 | 0.066666667 | 0.167320261 | 0 |
| 18 | *g__Hydrogenophaga* | 3 | 0.3333 | 0.066666667 | 0.210699588 | 0.017441077 |
| 19 | *g__Hyphomicrobium* | 4 | 0.1667 | 0.088888889 | 0.1 | 0.011111111 |
| 20 | *g__Hyphomonas* | 1 | 0 | 0.022222222 | 0.033333333 | 0 |
| 21 | *g__Ilumatobacter* | 4 | 0 | 0.088888889 | 0.277506775 | 0.159949495 |
| 22 | *g__Lacibacter* | 2 | 0 | 0.044444444 | 0.212668744 | 0.031313131 |
| 23 | *g__Luteolibacter* | 1 | 0 | 0.022222222 | 0.022222222 | 0 |
| 24 | *g__Mesorhizobium* | 3 | 1 | 0.066666667 | 0.234593356 | 0 |
| 25 | *g__Microbacterium* | 2 | 0 | 0.044444444 | 0.05 | 0.002020202 |
| 26 | *g__Mucilaginibacter* | 2 | 1 | 0.044444444 | 0.066666667 | 0 |
| 27 | *g__norank_f__A4b* | 7 | 0.381 | 0.155555556 | 0.288045007 | 0.061405323 |
| 28 | *g__norank_f__Caldilineaceae* | 3 | 1 | 0.066666667 | 0.167320261 | 0 |
| 29 | *g__norank_f__Microscillaceae* | 2 | 1 | 0.044444444 | 0.061538462 | 0 |
| 30 | *g__norank_f__Vicinamibacteraceae* | 3 | 0 | 0.066666667 | 0.197874396 | 0.03493266 |
| 31 | *g__norank_o__Microtrichales* | 6 | 0.5333 | 0.133333333 | 0.284444444 | 0.030092192 |
| 32 | *g__norank_o__Saccharimonadales* | 1 | 0 | 0.022222222 | 0.15585997 | 0 |
| 33 | *g__Pedobacter* | 1 | 0 | | | |
| 34 | *g__Pedomicrobium* | 4 | 0.8333 | 0.088888889 | 0.220927724 | 0.001214526 |
| 35 | *g__Planococcus* | 5 | 0.4 | 0.111111111 | 0.234593356 | 0.0265865 |
| 36 | *g__Pseudomonas* | 4 | 0.5 | 0.088888889 | 0.212668744 | 0.087878788 |

| | | | | | | |
|---|---|---|---|---|---|---|
| 37 | *g__Rhodobacter* | 5 | 0.3 | 0.111111111 | 0.277506775 | 0.122667949 |
| 38 | *g__Rosenbergiella* | 5 | 0.3 | 0.111111111 | 0.22530253 | 0.09364438 |
| 39 | *g__Sphingobium* | 4 | 0.6667 | 0.088888889 | 0.25006105 | 0.004242424 |
| 40 | *g__Staphylococcus* | 3 | 1 | 0.066666667 | 0.175042735 | 0 |
| 41 | *g__Tahibacter* | 2 | 1 | 0.044444444 | 0.066666667 | 0 |
| 42 | *g__unclassified_c__Alphaproteobacteria* | 8 | 0.2857 | 0.177777778 | 0.291737892 | 0.115108225 |
| 43 | *g__unclassified_f__Comamonadaceae* | 4 | 0.3333 | 0.088888889 | 0.258585859 | 0.040856983 |
| 44 | *g__unclassified_f__Enterobacteriaceae* | 3 | 1 | 0.066666667 | 0.175042735 | 0 |
| 45 | *g__unclassified_f__Rhizobiaceae* | 4 | 0.8333 | 0.088888889 | 0.232199546 | 0.002542088 |
| 46 | *g__unclassified_f__Rhodobacteraceae* | 2 | 0 | 0.044444444 | 0.220927724 | 0.006835017 |

**Table 5** species correlation network of bacteria in root of E+ plants.

| Node_ID | node_name | Degree | Custering | Degree_Centrality | Closeness_Centrality | Betweenness_Centrality |
|---|---|---|---|---|---|---|
| 1 | *g__Acinetobacter* | 8 | 1 | 0.210526316 | 0.210526316 | 0 |
| 2 | *g__Actinoplanes* | 3 | 1 | 0.078947368 | 0.078947368 | 0 |
| 3 | *g__Aeromicrobium* | 3 | 1 | 0.078947368 | 0.078947368 | 0 |
| 4 | *g__Allorhizobium-Neorhizobium-Pararhizobium-Rhizobium* | 1 | 0 | 0.026315789 | 0.026315789 | 0 |
| 5 | *g__Aquabacterium* | 6 | 1 | 0.157894737 | 0.157894737 | 0 |
| 6 | *g__Azospirillum* | 1 | 0 | 0.026315789 | 0.026315789 | 0 |
| 7 | *g__Bacillus* | 8 | 1 | 0.210526316 | 0.210526316 | 0 |
| 8 | *g__Bosea* | 1 | 0 | 0.026315789 | 0.026315789 | 0 |
| 9 | *g__Cellvibrio* | 6 | 1 | 0.157894737 | 0.157894737 | 0 |
| 10 | *g__Clostridium_sensu_stricto_1* | 3 | 1 | 0.078947368 | 0.078947368 | 0 |

| | | | | | | |
|---|---|---|---|---|---|---|
| 11 | *g__Comamonas* | 8 | 1 | 0.210526316 | 0.210526316 | 0 |
| 12 | *g__Delftia* | 4 | 1 | 0.105263158 | 0.105263158 | 0 |
| 13 | *g__Devosia* | 1 | 0 | 0.026315789 | 0.026315789 | 0 |
| 14 | *g__Emticicia* | 6 | 1 | 0.157894737 | 0.157894737 | 0 |
| 15 | *g__Escherichia-Shigella* | 8 | 1 | 0.210526316 | 0.210526316 | 0 |
| 16 | *g__Flavobacterium* | 1 | 0 | 0.026315789 | 0.026315789 | 0 |
| 17 | *g__Herpetosiphon* | 4 | 1 | 0.105263158 | 0.105263158 | 0 |
| 18 | *g__Hydrogenophaga* | 1 | 0 | 0.026315789 | 0.026315789 | 0 |
| 19 | *g__Ilumatobacter* | 1 | 0 | 0.026315789 | 0.026315789 | 0 |
| 20 | *g__Luteolibacter* | 3 | 1 | 0.078947368 | 0.078947368 | 0 |
| 21 | *g__Microbacterium* | 3 | 1 | 0.078947368 | 0.078947368 | 0 |
| 22 | *g__Microcoleus_PCC-7113* | 8 | 1 | 0.210526316 | 0.210526316 | 0 |
| 23 | *g__norank_f__BIrii41* | 4 | 1 | 0.105263158 | 0.105263158 | 0 |
| 24 | *g__norank_f__Caldilineaceae* | 4 | 1 | 0.105263158 | 0.105263158 | 0 |
| 25 | *g__norank_f__Microscillaceae* | 6 | 1 | 0.157894737 | 0.157894737 | 0 |
| 26 | *g__norank_f__Spirosomaceae* | 3 | 1 | 0.078947368 | 0.078947368 | 0 |
| 27 | *g__norank_f__Vicinamibacteraceae* | 4 | 1 | 0.105263158 | 0.105263158 | 0 |
| 28 | *g__Pedomicrobium* | 3 | 1 | 0.078947368 | 0.078947368 | 0 |
| 29 | *g__Pseudomonas* | 1 | 0 | 0.026315789 | 0.026315789 | 0 |
| 30 | *g__Rheinheimera* | 6 | 1 | 0.157894737 | 0.157894737 | 0 |
| 31 | *g__Rhodobacter* | 1 | 0 | 0.026315789 | 0.026315789 | 0 |
| 32 | *g__Rhodococcus* | 8 | 1 | 0.210526316 | 0.210526316 | 0 |
| 33 | *g__Rosenbergiella* | 8 | 1 | 0.210526316 | 0.210526316 | 0 |
| 34 | *g__Staphylococcus* | 8 | 1 | 0.210526316 | 0.210526316 | 0 |
| 35 | *g__SWB02* | 6 | 1 | 0.157894737 | 0.157894737 | 0 |

| | | | | | | |
|---|---|---|---|---|---|---|
| 36 | *g__Tahibacter* | 6 | 1 | 0.157894737 | 0.157894737 | 0 |
| 37 | *g__unclassified_f__Enterobacteriaceae* | 8 | 1 | 0.210526316 | 0.210526316 | 0 |
| 38 | *g__unclassified_f__Rhizobiaceae* | 3 | 1 | 0.078947368 | 0.078947368 | 0 |
| 39 | *g__unclassified_f__Rhodobacteraceae* | 1 | 0 | 0.026315789 | 0.026315789 | 0 |

**Table 6** species correlation network of bacteria in root of E- plants.

| Node_ID | node_name | Degree | Clustering | Degree_Centrality | Closeness_Centrality | Betweenness_Centrality |
|---|---|---|---|---|---|---|
| 1 | *g__Acidibacter* | 4 | 1 | 0.102564103 | 0.102564103 | 0 |
| 2 | *g__Acidovorax* | 1 | 0 | 0.025641026 | 0.025641026 | 0 |
| 3 | *g__Actinoplanes* | 1 | 0 | 0.025641026 | 0.025641026 | 0 |
| 4 | *g__Aliihoeflea* | 7 | 1 | 0.179487179 | 0.179487179 | 0 |
| 5 | *g__Bauldia* | 2 | 1 | 0.051282051 | 0.051282051 | 0 |
| 6 | *g__Bdellovibrio* | 7 | 1 | 0.179487179 | 0.179487179 | 0 |
| 7 | *g__Blastomonas* | 5 | 1 | 0.128205128 | 0.128205128 | 0 |
| 8 | *g__Brevundimonas* | 2 | 1 | 0.051282051 | 0.051282051 | 0 |
| 9 | *g__Chryseobacterium* | 7 | 1 | 0.179487179 | 0.179487179 | 0 |
| 10 | *g__Cytophaga* | 1 | 0 | 0.025641026 | 0.025641026 | 0 |
| 11 | *g__Exiguobacterium* | 1 | 0 | 0.025641026 | 0.025641026 | 0 |
| 12 | *g__Flavihumibacter* | 1 | 0 | 0.025641026 | 0.025641026 | 0 |
| 13 | *g__Flavobacterium* | 1 | 0 | 0.025641026 | 0.025641026 | 0 |
| 14 | *g__Hirschia* | 5 | 1 | 0.128205128 | 0.128205128 | 0 |
| 15 | *g__Hydrogenophaga* | 1 | 0 | 0.025641026 | 0.025641026 | 0 |
| 16 | *g__Hyphomicrobium* | 1 | 0 | 0.025641026 | 0.025641026 | 0 |
| 17 | *g__Hyphomonas* | 4 | 1 | 0.102564103 | 0.102564103 | 0 |
| 18 | *g__Ilumatobacter* | 4 | 1 | 0.102564103 | 0.102564103 | 0 |

| | | | | | | |
|---|---|---|---|---|---|---|
| 19 | g__*Legionella* | 7 | 1 | 0.179487179 | 0.179487179 | 0 |
| 20 | g__*Luteolibacter* | 7 | 1 | 0.179487179 | 0.179487179 | 0 |
| 21 | g__*Mesorhizobium* | 5 | 1 | 0.128205128 | 0.128205128 | 0 |
| 22 | g__*Microbacterium* | 4 | 1 | 0.102564103 | 0.102564103 | 0 |
| 23 | g__*Mucilaginibacter* | 5 | 1 | 0.128205128 | 0.128205128 | 0 |
| 24 | g__*norank_f__Azospirillaceae* | 5 | 1 | 0.128205128 | 0.128205128 | 0 |
| 25 | g__*norank_f__Caldilineaceae* | 2 | 1 | 0.051282051 | 0.051282051 | 0 |
| 26 | g__*norank_f__Ilumatobacteraceae* | 5 | 1 | 0.128205128 | 0.128205128 | 0 |
| 27 | g__*norank_f__Microscillaceae* | 4 | 1 | 0.102564103 | 0.102564103 | 0 |
| 28 | g__*norank_f__Sandaracinaceae* | 7 | 1 | 0.179487179 | 0.179487179 | 0 |
| 29 | g__*norank_o__Microtrichales* | 5 | 1 | 0.128205128 | 0.128205128 | 0 |
| 30 | g__*norank_o__Saccharimonadales* | 1 | 0 | 0.025641026 | 0.025641026 | 0 |
| 31 | g__*OLB13* | 5 | 1 | 0.128205128 | 0.128205128 | 0 |
| 32 | g__*Pedobacter* | 5 | 1 | 0.128205128 | 0.128205128 | 0 |
| 33 | g__*Pedomicrobium* | 7 | 1 | 0.179487179 | 0.179487179 | 0 |
| 34 | g__*Planococcus* | 1 | 0 | 0.025641026 | 0.025641026 | 0 |
| 35 | g__*Pseudomonas* | 7 | 1 | 0.179487179 | 0.179487179 | 0 |
| 36 | g__*Pseudoxanthomonas* | 5 | 1 | 0.128205128 | 0.128205128 | 0 |
| 37 | g__*Rhodobacter* | 1 | 0 | 0.025641026 | 0.025641026 | 0 |
| 38 | g__*Sphingopyxis* | 5 | 1 | 0.128205128 | 0.128205128 | 0 |
| 39 | g__*Tahibacter* | 1 | 0 | 0.025641026 | 0.025641026 | 0 |
| 40 | g__*Terrimonas* | 5 | 1 | 0.128205128 | 0.128205128 | 0 |

**SUPPLEMENTAL FILE S2**

**Table 1** Coefficient and significance between node 1 and node 2 in correlation network of shoot of E+ and E- plants.

| Node1_Name | Node2_Name | Coefficient | P_value |
|---|---|---|---|
| *g__Azospirillum* | *g__Jeotgalibacillus* | -0.72281 | 0.042790387 |
| *g__unclassified_f__Rhodobacteraceae* | *g__Azospirillum* | 0.75292 | 0.031065869 |
| *g__Delftia* | *g__Azospirillum* | 0.7769 | 0.023323763 |
| *g__Hyphomonas* | *g__Bauldia* | 1 | 3.42E-48 |
| *g__norank_o__Microtrichales* | *g__Bauldia* | 1 | 3.42E-48 |
| *g__Brachybacterium* | *g__Bauldia* | 1 | 3.42E-48 |
| *g__norank_o__Microtrichales* | *g__Hyphomonas* | 1 | 3.42E-48 |
| *g__Brachybacterium* | *g__Hyphomonas* | 1 | 3.42E-48 |
| *g__Brachybacterium* | *g__norank_o__Microtrichales* | 1 | 3.42E-48 |
| *g__Acinetobacter* | *g__Exiguobacterium* | 0.76321 | 0.027574766 |
| *g__Paracoccus* | *g__Acinetobacter* | -0.88498 | 0.003483144 |
| *g__Paenarthrobacter* | *g__Paracoccus* | -0.77913 | 0.022671515 |
| *g__Gemmobacter* | *g__Paracoccus* | -0.76509 | 0.026965391 |
| *g__Aquabacterium* | *g__Paracoccus* | -0.76509 | 0.026965391 |
| *g__Herpetosiphon* | *g__Pseudoxanthomonas* | 0.75593 | 0.030019745 |
| *g__Paenarthrobacter* | *g__Devosia* | -0.71393 | 0.046687099 |
| *g__Planococcus* | *g__Paenarthrobacter* | -0.72856 | 0.040372376 |
| *g__Gemmobacter* | *g__Paenarthrobacter* | 0.80013 | 0.017087879 |
| *g__Aquabacterium* | *g__Paenarthrobacter* | 0.80013 | 0.017087879 |
| *g__Reyranella* | *g__norank_p__WPS-2* | 1 | 3.42E-48 |
| *g__Porphyrobacter* | *g__norank_p__WPS-2* | 0.75593 | 0.030019745 |
| *g__norank_f__67-14* | *g__norank_p__WPS-2* | 1 | 3.42E-48 |

| | | | |
|---|---|---|---|
| *g__Reyranella* | *g__norank_f__67-14* | 1 | 3.42E-48 |
| *g__Porphyrobacter* | *g__norank_f__67-14* | 0.75593 | 0.030019745 |
| *g__Porphyrobacter* | *g__Reyranella* | 0.75593 | 0.030019745 |
| *g__Novosphingobium* | *g__Porphyrobacter* | 0.81892 | 0.012900447 |
| *g__Enterobacter* | *g__Planococcus* | -0.82479 | 0.011742396 |
| *g__Rhodococcus* | *g__Planococcus* | -0.86603 | 0.00542395 |
| *g__Aeromicrobium* | *g__Planococcus* | -0.86603 | 0.00542395 |
| *g__Gemmobacter* | *g__Planococcus* | -0.75593 | 0.030019745 |
| *g__Aquabacterium* | *g__Planococcus* | -0.75593 | 0.030019745 |
| *g__Rhodococcus* | *g__Enterobacter* | 0.97619 | 3.31E-05 |
| *g__Aeromicrobium* | *g__Enterobacter* | 0.97619 | 3.31E-05 |
| *g__Gemmobacter* | *g__Enterobacter* | 0.80013 | 0.017087879 |
| *g__Aquabacterium* | *g__Enterobacter* | 0.80013 | 0.017087879 |
| *g__Aeromicrobium* | *g__Rhodococcus* | 1 | 0 |
| *g__Gemmobacter* | *g__Rhodococcus* | 0.87287 | 0.004659215 |
| *g__Aquabacterium* | *g__Rhodococcus* | 0.87287 | 0.004659215 |
| *g__Gemmobacter* | *g__Aeromicrobium* | 0.87287 | 0.004659215 |
| *g__Aquabacterium* | *g__Aeromicrobium* | 0.87287 | 0.004659215 |
| *g__unclassified_f__Rhodobacteraceae* | *g__Gemmobacter* | 0.80013 | 0.017087879 |
| *g__Aquabacterium* | *g__Gemmobacter* | 1 | 0 |
| *g__unclassified_f__Rhodobacteraceae* | *g__Aquabacterium* | 0.80013 | 0.017087879 |
| *g__Cellvibrio* | *g__Verrucomicrobium* | 1 | 3.42E-48 |
| *g__Mycobacterium* | *g__Verrucomicrobium* | 1 | 3.42E-48 |
| *g__Flavihumibacter* | *g__Verrucomicrobium* | 1 | 3.42E-48 |
| *g__Chryseobacterium* | *g__Verrucomicrobium* | 0.75593 | 0.030019745 |

| | | | |
|---|---|---|---|
| *g__Mycobacterium* | *g__Cellvibrio* | 1 | 3.42E-48 |
| *g__Flavihumibacter* | *g__Cellvibrio* | 1 | 3.42E-48 |
| *g__Chryseobacterium* | *g__Cellvibrio* | 0.75593 | 0.030019745 |
| *g__Flavihumibacter* | *g__Mycobacterium* | 1 | 3.42E-48 |
| *g__Chryseobacterium* | *g__Mycobacterium* | 0.75593 | 0.030019745 |
| *g__Chryseobacterium* | *g__Flavihumibacter* | 0.75593 | 0.030019745 |
| *g__Delftia* | *g__Chryseobacterium* | 0.875 | 0.004436493 |
| *g__unclassified_f__Rhizobiaceae* | *g__Chryseobacterium* | 0.98974 | 2.68E-06 |
| *g__unclassified_f__Rhizobiaceae* | *g__Delftia* | 0.86603 | 0.00542395 |
| *g__Flavobacterium* | *g__Delftia* | 0.79579 | 0.018162034 |
| *g__Bosea* | *g__Novosphingobium* | 0.75292 | 0.031065869 |
| *g__Pseudomonas* | *g__Allorhizobium-Neorhizobium-Pararhizobium-Rhizobium* | 0.83032 | 0.010712066 |
| *g__Bosea* | *g__Allorhizobium-Neorhizobium-Pararhizobium-Rhizobium* | 0.79195 | 0.019147899 |
| *g__Bosea* | *g__Pseudomonas* | 0.8134 | 0.014055128 |
| *g__Bdellovibrio* | *g__unclassified_o__Rhizobiales* | 1 | 3.42E-48 |
| *g__Hydrogenophaga* | *g__unclassified_o__Rhizobiales* | 0.75593 | 0.030019745 |
| *g__Brevundimonas* | *g__unclassified_o__Rhizobiales* | 1 | 3.42E-48 |
| *g__norank_f__Solirubrobacteraceae* | *g__unclassified_o__Rhizobiales* | 1 | 3.42E-48 |
| *g__Bdellovibrio* | *g__Brevundimonas* | 1 | 3.42E-48 |
| *g__Hydrogenophaga* | *g__Brevundimonas* | 0.75593 | 0.030019745 |
| *g__norank_f__Solirubrobacteraceae* | *g__Brevundimonas* | 1 | 3.42E-48 |
| *g__Bdellovibrio* | *g__norank_f__Solirubrobacteraceae* | 1 | 3.42E-48 |
| *g__Hydrogenophaga* | *g__norank_f__Solirubrobacteraceae* | 0.75593 | 0.030019745 |
| *g__Hydrogenophaga* | *g__Bdellovibrio* | 0.75593 | 0.030019745 |
| *g__Acidovorax* | *g__Hydrogenophaga* | 0.76835 | 0.025927834 |

| | | | |
|---|---|---|---|
| *g__Microbacterium* | *g__Acidovorax* | 0.74512 | 0.033884436 |
| *g__unclassified_f__Sphingomonadaceae* | *g__Sphingobium* | 1 | 3.42E-48 |

**Table 2** Coefficient and significance between node 1 and node 2 in correlation network of shoot of E+ plants.

| Node1_Name | Node2_Name | Coefficient | P_value |
|---|---|---|---|
| *g__Acinetobacter* | *g__Paenarthrobacter* | 1 | 0 |
| *g__Actinoplanes* | *g__Enterobacter* | -1 | 0 |
| *g__Aeromicrobium* | *g__Rhodococcus* | 1 | 0 |
| *g__Bdellovibrio* | *g__Pseudoxanthomonas* | 1 | 0 |
| *g__Bdellovibrio* | *g__Sphingomonas* | 1 | 0 |
| *g__Bdellovibrio* | *g__Porphyrobacter* | 1 | 0 |
| *g__Bdellovibrio* | *g__unclassified_o__Rhizobiales* | 1 | 0 |
| *g__Bdellovibrio* | *g__Brevundimonas* | 1 | 0 |
| *g__Bdellovibrio* | *g__norank_f__Solirubrobacteraceae* | 1 | 0 |
| *g__Brevundimonas* | *g__Pseudoxanthomonas* | 1 | 0 |
| *g__Brevundimonas* | *g__Sphingomonas* | 1 | 0 |
| *g__Brevundimonas* | *g__Porphyrobacter* | 1 | 0 |
| *g__Brevundimonas* | *g__unclassified_o__Rhizobiales* | 1 | 0 |
| *g__Cellvibrio* | *g__Rhodobacter* | -1 | 0 |
| *g__Cellvibrio* | *g__Verrucomicrobium* | 1 | 0 |
| *g__Dyadobacter* | *g__Azospirillum* | 1 | 0 |
| *g__Dyadobacter* | *g__norank_c__Sericytochromatia* | 1 | 0 |
| *g__Dyadobacter* | *g__unclassified_f__Flavobacteriaceae* | 1 | 0 |
| *g__Dyadobacter* | *g__unclassified_f__Microbacteriaceae* | 1 | 0 |
| *g__Flavihumibacter* | *g__Rhodobacter* | -1 | 0 |

| | | | |
|---|---|---|---|
| *g__Flavihumibacter* | *g__Verrucomicrobium* | 1 | 0 |
| *g__Flavihumibacter* | *g__Cellvibrio* | 1 | 0 |
| *g__Flavihumibacter* | *g__Mycobacterium* | 1 | 0 |
| *g__Gemmobacter* | *g__Aquabacterium* | 1 | 0 |
| *g__Mycobacterium* | *g__Rhodobacter* | -1 | 0 |
| *g__Mycobacterium* | *g__Verrucomicrobium* | 1 | 0 |
| *g__Mycobacterium* | *g__Cellvibrio* | 1 | 0 |
| *g__norank_c__Sericytochromatia* | *g__Azospirillum* | 1 | 0 |
| *g__norank_f__Solirubrobacteraceae* | *g__Pseudoxanthomonas* | 1 | 0 |
| *g__norank_f__Solirubrobacteraceae* | *g__Sphingomonas* | 1 | 0 |
| *g__norank_f__Solirubrobacteraceae* | *g__Porphyrobacter* | 1 | 0 |
| *g__norank_f__Solirubrobacteraceae* | *g__unclassified_o__Rhizobiales* | 1 | 0 |
| *g__norank_f__Solirubrobacteraceae* | *g__Brevundimonas* | 1 | 0 |
| *g__Novosphingobium* | *g__Aquabacterium* | 1 | 0 |
| *g__Novosphingobium* | *g__Gemmobacter* | 1 | 0 |
| *g__Paracoccus* | *g__Pseudomonas* | -1 | 0 |
| *g__Porphyrobacter* | *g__Pseudoxanthomonas* | 1 | 0 |
| *g__Porphyrobacter* | *g__Sphingomonas* | 1 | 0 |
| *g__Sphingomonas* | *g__Pseudoxanthomonas* | 1 | 0 |
| *g__unclassified_f__Flavobacteriaceae* | *g__Azospirillum* | 1 | 0 |
| *g__unclassified_f__Flavobacteriaceae* | *g__norank_c__Sericytochromatia* | 1 | 0 |
| *g__unclassified_f__Microbacteriaceae* | *g__Azospirillum* | 1 | 0 |
| *g__unclassified_f__Microbacteriaceae* | *g__norank_c__Sericytochromatia* | 1 | 0 |
| *g__unclassified_f__Microbacteriaceae* | *g__unclassified_f__Flavobacteriaceae* | 1 | 0 |
| *g__unclassified_f__Rhizobiaceae* | *g__Aquabacterium* | -1 | 0 |

| | | | |
|---|---|---|---|
| *g__unclassified_f__Rhizobiaceae* | *g__Gemmobacter* | -1 | 0 |
| *g__unclassified_f__Rhizobiaceae* | *g__Novosphingobium* | -1 | 0 |
| *g__unclassified_f__Sphingomonadaceae* | *g__Sphingobium* | 1 | 0 |
| *g__unclassified_o__Rhizobiales* | *g__Pseudoxanthomonas* | 1 | 0 |
| *g__unclassified_o__Rhizobiales* | *g__Sphingomonas* | 1 | 0 |
| *g__unclassified_o__Rhizobiales* | *g__Porphyrobacter* | 1 | 0 |
| *g__Verrucomicrobium* | *g__Rhodobacter* | -1 | 0 |

**Table 3** Coefficient and significance between node 1 and node 2 in correlation network of shoot of E- plants.

| Node1_Name | Node2_Name | Coefficient | P_value |
|---|---|---|---|
| *g__Actinoplanes* | *g__Pseudoxanthomonas* | 1 | 0 |
| *g__Allorhizobium-Neorhizobium-Pararhizobium-Rhizobium* | *g__Flavobacterium* | 1 | 0 |
| *g__Brachybacterium* | *g__Bauldia* | 1 | 0 |
| *g__Brachybacterium* | *g__Hyphomonas* | 1 | 0 |
| *g__Brachybacterium* | *g__Paenarthrobacter* | 1 | 0 |
| *g__Brachybacterium* | *g__norank_o__Microtrichales* | 1 | 0 |
| *g__Herpetosiphon* | *g__Pseudoxanthomonas* | 1 | 0 |
| *g__Herpetosiphon* | *g__Actinoplanes* | 1 | 0 |
| *g__Hyphomonas* | *g__Bauldia* | 1 | 0 |
| *g__norank_f__67-14* | *g__norank_p__WPS-2* | 1 | 0 |
| *g__norank_f__67-14* | *g__Porphyrobacter* | 1 | 0 |
| *g__norank_f__67-14* | *g__Novosphingobium* | 1 | 0 |
| *g__norank_f__67-14* | *g__Reyranella* | 1 | 0 |
| *g__norank_f__67-14* | *g__unclassified_f__Microbacteriaceae* | 1 | 0 |
| *g__norank_o__Microtrichales* | *g__Bauldia* | 1 | 0 |

| | | | |
|---|---|---|---|
| *g__norank_o__Microtrichales* | *g__Hyphomonas* | 1 | 0 |
| *g__norank_o__Microtrichales* | *g__Paenarthrobacter* | 1 | 0 |
| *g__Novosphingobium* | *g__norank_p__WPS-2* | 1 | 0 |
| *g__Novosphingobium* | *g__Porphyrobacter* | 1 | 0 |
| *g__Paenarthrobacter* | *g__Bauldia* | 1 | 0 |
| *g__Paenarthrobacter* | *g__Hyphomonas* | 1 | 0 |
| *g__Paracoccus* | *g__Exiguobacterium* | -1 | 0 |
| *g__Porphyrobacter* | *g__norank_p__WPS-2* | 1 | 0 |
| *g__Pseudomonas* | *g__Exiguobacterium* | -1 | 0 |
| *g__Pseudomonas* | *g__Paracoccus* | 1 | 0 |
| *g__Reyranella* | *g__norank_p__WPS-2* | 1 | 0 |
| *g__Reyranella* | *g__Porphyrobacter* | 1 | 0 |
| *g__Reyranella* | *g__Novosphingobium* | 1 | 0 |
| *g__Rhodobacter* | *g__Jeotgalibacillus* | -1 | 0 |
| *g__unclassified_f__Microbacteriaceae* | *g__norank_p__WPS-2* | 1 | 0 |
| *g__unclassified_f__Microbacteriaceae* | *g__Porphyrobacter* | 1 | 0 |
| *g__unclassified_f__Microbacteriaceae* | *g__Novosphingobium* | 1 | 0 |
| *g__unclassified_f__Microbacteriaceae* | *g__Reyranella* | 1 | 0 |

**Table 4** Coefficient and significance between node 1 and node 2 in correlation network of root of E+ and E- plants.

| Node1_Name | Node2_Name | Coefficient | P_value |
|---|---|---|---|
| *g__Acidibacter* | *g__unclassified_c__Alphaproteobacteria* | 0.7746 | 0.024008197 |
| *g__Acidibacter* | *g__norank_o__Microtrichales* | 0.74536 | 0.033797788 |
| *g__Acidibacter* | *g__norank_f__A4b* | 0.73771 | 0.036702195 |
| *g__Acidibacter* | *g__Ilumatobacter* | -0.72528 | 0.041741614 |

| | | | |
|---|---|---|---|
| *g__Acidibacter* | *g__Rhodobacter* | 0.79837 | 0.017518391 |
| *g__Acidovorax* | *g__norank_f__Caldilineaceae* | 0.86066 | 0.006075947 |
| *g__Acidovorax* | *g__Herpetosiphon* | 0.86066 | 0.006075947 |
| *g__Acinetobacter* | *g__Rosenbergiella* | 0.75593 | 0.030019745 |
| *g__Allorhizobium-Neorhizobium-Pararhizobium-Rhizobium* | *g__Exiguobacterium* | 0.88002 | 0.003939096 |
| *g__Azospirillum* | *g__Lacibacter* | 0.84855 | 0.00772823 |
| *g__Bdellovibrio* | *g__Hyphomicrobium* | 0.75724 | 0.029570257 |
| *g__Bosea* | *g__Devosia* | 0.72729 | 0.040900739 |
| *g__Bosea* | *g__norank_f__Vicinamibacteraceae* | 0.71275 | 0.047221675 |
| *g__Bosea* | *g__unclassified_f__Rhodobacteraceae* | 0.7408 | 0.035512126 |
| *g__Cellvibrio* | *g__Flavobacterium* | 0.76047 | 0.02848113 |
| *g__Cellvibrio* | *g__norank_f__Microscillaceae* | 1 | 0 |
| *g__Cytophaga* | *g__Rhodobacter* | 0.78896 | 0.019936718 |
| *g__Devosia* | *g__Sphingobium* | -0.85023 | 0.007483552 |
| *g__Enterobacter* | *g__Devosia* | -0.82974 | 0.010817007 |
| *g__Enterobacter* | *g__unclassified_c__Alphaproteobacteria* | -0.75593 | 0.030019745 |
| *g__Flavihumibacter* | *g__Planococcus* | 0.71393 | 0.046687099 |
| *g__Flavihumibacter* | *g__Hydrogenophaga* | 0.73044 | 0.03960299 |
| *g__Herpetosiphon* | *g__norank_f__Caldilineaceae* | 1 | 0 |
| *g__Hydrogenophaga* | *g__Rosenbergiella* | -0.7178 | 0.044961469 |
| *g__Hyphomicrobium* | *g__Flavobacterium* | 0.73734 | 0.036845749 |
| *g__Hyphomonas* | *g__Microbacterium* | 0.75593 | 0.030019745 |
| *g__Ilumatobacter* | *g__Comamonas* | -0.7078 | 0.049501734 |
| *g__Lacibacter* | *g__unclassified_c__Alphaproteobacteria* | -0.75 | 0.032104492 |
| *g__Luteolibacter* | *g__Chryseobacterium* | -0.72281 | 0.042790387 |

| | | | |
|---|---|---|---|
| g__*Mesorhizobium* | g__*Devosia* | 0.73687 | 0.037032171 |
| g__*Mesorhizobium* | g__*norank_o__Microtrichales* | 0.74536 | 0.033797788 |
| g__*Mesorhizobium* | g__*norank_f__A4b* | 0.73771 | 0.036702195 |
| g__*Microbacterium* | g__*Exiguobacterium* | 0.78585 | 0.020778519 |
| g__*Mucilaginibacter* | g__*Hyphomicrobium* | 0.70711 | 0.049825263 |
| g__*norank_f__A4b* | g__*Sphingobium* | -0.73771 | 0.036702195 |
| g__*norank_f__A4b* | g__*Devosia* | 0.75267 | 0.031155298 |
| g__*norank_f__A4b* | g__*norank_o__Microtrichales* | 0.98974 | 2.68E-06 |
| g__*norank_f__Microscillaceae* | g__*Flavobacterium* | 0.76047 | 0.02848113 |
| g__*norank_f__Vicinamibacteraceae* | g__*Comamonas* | -0.72281 | 0.042790387 |
| g__*norank_o__Microtrichales* | g__*Sphingobium* | -0.74536 | 0.033797788 |
| g__*norank_o__Microtrichales* | g__*Devosia* | 0.76047 | 0.02848113 |
| g__*norank_o__Saccharimonadales* | g__*norank_f__Vicinamibacteraceae* | -0.74066 | 0.035566022 |
| g__*Pedobacter* | g__*norank_f__A4b* | 0.75593 | 0.030019745 |
| g__*Pedomicrobium* | g__*unclassified_c__Alphaproteobacteria* | 0.7769 | 0.023323763 |
| g__*Pedomicrobium* | g__*Enterobacter* | -0.90351 | 0.002086622 |
| g__*Pedomicrobium* | g__*unclassified_f__Rhizobiaceae* | 0.77816 | 0.022952535 |
| g__*Planococcus* | g__*unclassified_c__Alphaproteobacteria* | 0.83333 | 0.01017554 |
| g__*Planococcus* | g__*Enterobacter* | -0.71393 | 0.046687099 |
| g__*Planococcus* | g__*Pedomicrobium* | 0.75698 | 0.029659759 |
| g__*Pseudomonas* | g__*Ilumatobacter* | 0.72791 | 0.040642489 |
| g__*Pseudomonas* | g__*norank_f__Caldilineaceae* | 0.72036 | 0.043844081 |
| g__*Pseudomonas* | g__*Herpetosiphon* | 0.72036 | 0.043844081 |
| g__*Pseudomonas* | g__*Acidovorax* | 0.91061 | 0.001668307 |
| g__*Rhodobacter* | g__*norank_o__Microtrichales* | 0.76509 | 0.026965391 |

| | | | |
|---|---|---|---|
| *g__Rhodobacter* | *g__norank_f__A4b* | 0.75724 | 0.0295687 |
| *g__Rhodobacter* | *g__Rosenbergiella* | -0.77302 | 0.024483883 |
| *g__Staphylococcus* | *g__Rosenbergiella* | 0.75593 | 0.030019745 |
| *g__Staphylococcus* | *g__Acinetobacter* | 1 | 3.42E-48 |
| *g__Tahibacter* | *g__Hyphomicrobium* | 0.76064 | 0.028424575 |
| *g__Tahibacter* | *g__Mucilaginibacter* | 0.95618 | 0.000203465 |
| *g__unclassified_c__Alphaproteobacteria* | *g__Sphingobium* | -0.7746 | 0.024008197 |
| *g__unclassified_c__Alphaproteobacteria* | *g__Devosia* | 0.76835 | 0.025927834 |
| *g__unclassified_f__Comamonadaceae* | *g__Planococcus* | 0.7698 | 0.025473928 |
| *g__unclassified_f__Comamonadaceae* | *g__Hydrogenophaga* | 0.76509 | 0.026965391 |
| *g__unclassified_f__Comamonadaceae* | *g__Acidibacter* | 0.74536 | 0.033797788 |
| *g__unclassified_f__Comamonadaceae* | *g__Flavihumibacter* | 0.80013 | 0.017087879 |
| *g__unclassified_f__Enterobacteriaceae* | *g__Rosenbergiella* | 0.75593 | 0.030019745 |
| *g__unclassified_f__Enterobacteriaceae* | *g__Acinetobacter* | 1 | 3.42E-48 |
| *g__unclassified_f__Enterobacteriaceae* | *g__Staphylococcus* | 1 | 3.42E-48 |
| *g__unclassified_f__Rhizobiaceae* | *g__Devosia* | 0.76434 | 0.027207247 |
| *g__unclassified_f__Rhizobiaceae* | *g__unclassified_c__Alphaproteobacteria* | 0.73598 | 0.037380231 |
| *g__unclassified_f__Rhizobiaceae* | *g__Enterobacter* | -0.85592 | 0.006692551 |
| *g__unclassified_f__Rhodobacteraceae* | *g__Ilumatobacter* | 0.78514 | 0.020974301 |

**Table 5** Coefficient and significance between node 1 and node 2 in correlation network of root of E+ plants.

| Node1_Name | Node2_Name | Coefficient | P_value |
|---|---|---|---|
| *g__Actinoplanes* | *g__unclassified_f__Rhizobiaceae* | 1 | 0 |
| *g__Actinoplanes* | *g__Pedomicrobium* | 1 | 0 |
| *g__Aeromicrobium* | *g__Luteolibacter* | 1 | 0 |

| | | | |
|---|---|---|---|
| *g__Aeromicrobium* | *g__Clostridium_sensu_stricto_1* | 1 | 0 |
| *g__Aeromicrobium* | *g__Microbacterium* | 1 | 0 |
| *g__Allorhizobium-Neorhizobium-Pararhizobium-Rhizobium* | *g__Azospirillum* | 1 | 0 |
| *g__Aquabacterium* | *g__Tahibacter* | 1 | 0 |
| *g__Aquabacterium* | *g__Cellvibrio* | 1 | 0 |
| *g__Bacillus* | *g__Acinetobacter* | 1 | 0 |
| *g__Bacillus* | *g__Rosenbergiella* | 1 | 0 |
| *g__Bacillus* | *g__unclassified_f__Enterobacteriaceae* | 1 | 0 |
| *g__Bacillus* | *g__Escherichia-Shigella* | 1 | 0 |
| *g__Bacillus* | *g__Comamonas* | 1 | 0 |
| *g__Bacillus* | *g__Staphylococcus* | 1 | 0 |
| *g__Cellvibrio* | *g__Tahibacter* | 1 | 0 |
| *g__Clostridium_sensu_stricto_1* | *g__Luteolibacter* | 1 | 0 |
| *g__Comamonas* | *g__Acinetobacter* | 1 | 0 |
| *g__Comamonas* | *g__Rosenbergiella* | 1 | 0 |
| *g__Comamonas* | *g__unclassified_f__Enterobacteriaceae* | 1 | 0 |
| *g__Comamonas* | *g__Escherichia-Shigella* | 1 | 0 |
| *g__Emticicia* | *g__Tahibacter* | 1 | 0 |
| *g__Emticicia* | *g__Cellvibrio* | 1 | 0 |
| *g__Emticicia* | *g__Aquabacterium* | 1 | 0 |
| *g__Emticicia* | *g__Rheinheimera* | 1 | 0 |
| *g__Emticicia* | *g__SWB02* | 1 | 0 |
| *g__Emticicia* | *g__norank_f__Microscillaceae* | 1 | 0 |
| *g__Escherichia-Shigella* | *g__Acinetobacter* | 1 | 0 |
| *g__Escherichia-Shigella* | *g__Rosenbergiella* | 1 | 0 |

| | | | |
|---|---|---|---|
| *g__Escherichia-Shigella* | *g__unclassified_f__Enterobacteriaceae* | 1 | 0 |
| *g__Herpetosiphon* | *g__Delftia* | 1 | 0 |
| *g__Herpetosiphon* | *g__norank_f__BIrii41* | 1 | 0 |
| *g__Hydrogenophaga* | *g__Ilumatobacter* | 1 | 0 |
| *g__Microbacterium* | *g__Luteolibacter* | 1 | 0 |
| *g__Microbacterium* | *g__Clostridium_sensu_stricto_1* | 1 | 0 |
| *g__Microcoleus_PCC-7113* | *g__Acinetobacter* | 1 | 0 |
| *g__Microcoleus_PCC-7113* | *g__Rosenbergiella* | 1 | 0 |
| *g__Microcoleus_PCC-7113* | *g__unclassified_f__Enterobacteriaceae* | 1 | 0 |
| *g__Microcoleus_PCC-7113* | *g__Escherichia-Shigella* | 1 | 0 |
| *g__Microcoleus_PCC-7113* | *g__Comamonas* | 1 | 0 |
| *g__Microcoleus_PCC-7113* | *g__Staphylococcus* | 1 | 0 |
| *g__Microcoleus_PCC-7113* | *g__Bacillus* | 1 | 0 |
| *g__Microcoleus_PCC-7113* | *g__Rhodococcus* | 1 | 0 |
| *g__norank_f__BIrii41* | *g__Delftia* | 1 | 0 |
| *g__norank_f__Caldilineaceae* | *g__Delftia* | 1 | 0 |
| *g__norank_f__Caldilineaceae* | *g__norank_f__BIrii41* | 1 | 0 |
| *g__norank_f__Caldilineaceae* | *g__Herpetosiphon* | 1 | 0 |
| *g__norank_f__Caldilineaceae* | *g__norank_f__Vicinamibacteraceae* | 1 | 0 |
| *g__norank_f__Microscillaceae* | *g__Tahibacter* | 1 | 0 |
| *g__norank_f__Microscillaceae* | *g__Cellvibrio* | 1 | 0 |
| *g__norank_f__Microscillaceae* | *g__Aquabacterium* | 1 | 0 |
| *g__norank_f__Microscillaceae* | *g__Rheinheimera* | 1 | 0 |
| *g__norank_f__Microscillaceae* | *g__SWB02* | 1 | 0 |
| *g__norank_f__Spirosomaceae* | *g__unclassified_f__Rhizobiaceae* | 1 | 0 |

| | | | |
|---|---|---|---|
| *g__norank_f__Spirosomaceae* | *g__Pedomicrobium* | 1 | 0 |
| *g__norank_f__Spirosomaceae* | *g__Actinoplanes* | 1 | 0 |
| *g__norank_f__Vicinamibacteraceae* | *g__Delftia* | 1 | 0 |
| *g__norank_f__Vicinamibacteraceae* | *g__norank_f__BIrii41* | 1 | 0 |
| *g__norank_f__Vicinamibacteraceae* | *g__Herpetosiphon* | 1 | 0 |
| *g__Pedomicrobium* | *g__unclassified_f__Rhizobiaceae* | 1 | 0 |
| *g__Pseudomonas* | *g__Devosia* | 1 | 0 |
| *g__Rheinheimera* | *g__Tahibacter* | 1 | 0 |
| *g__Rheinheimera* | *g__Cellvibrio* | 1 | 0 |
| *g__Rheinheimera* | *g__Aquabacterium* | 1 | 0 |
| *g__Rhodobacter* | *g__Flavobacterium* | 1 | 0 |
| *g__Rhodococcus* | *g__Acinetobacter* | 1 | 0 |
| *g__Rhodococcus* | *g__Rosenbergiella* | 1 | 0 |
| *g__Rhodococcus* | *g__unclassified_f__Enterobacteriaceae* | 1 | 0 |
| *g__Rhodococcus* | *g__Escherichia-Shigella* | 1 | 0 |
| *g__Rhodococcus* | *g__Comamonas* | 1 | 0 |
| *g__Rhodococcus* | *g__Staphylococcus* | 1 | 0 |
| *g__Rhodococcus* | *g__Bacillus* | 1 | 0 |
| *g__Rosenbergiella* | *g__Acinetobacter* | 1 | 0 |
| *g__Staphylococcus* | *g__Acinetobacter* | 1 | 0 |
| *g__Staphylococcus* | *g__Rosenbergiella* | 1 | 0 |
| *g__Staphylococcus* | *g__unclassified_f__Enterobacteriaceae* | 1 | 0 |
| *g__Staphylococcus* | *g__Escherichia-Shigella* | 1 | 0 |
| *g__Staphylococcus* | *g__Comamonas* | 1 | 0 |
| *g__SWB02* | *g__Tahibacter* | 1 | 0 |

| | | | |
|---|---|---|---|
| *g__SWB02* | *g__Cellvibrio* | 1 | 0 |
| *g__SWB02* | *g__Aquabacterium* | 1 | 0 |
| *g__SWB02* | *g__Rheinheimera* | 1 | 0 |
| *g__unclassified_f__Enterobacteriaceae* | *g__Acinetobacter* | 1 | 0 |
| *g__unclassified_f__Enterobacteriaceae* | *g__Rosenbergiella* | 1 | 0 |
| *g__unclassified_f__Rhodobacteraceae* | *g__Bosea* | 1 | 0 |

**Table 6** Coefficient and significance between node 1 and node 2 in correlation network of root of E- plants.

| Node1_Name | Node2_Name | Coefficient | P_value |
|---|---|---|---|
| *g__Acidibacter* | *g__Hyphomonas* | -1 | 0 |
| *g__Acidibacter* | *g__norank_f__Microscillaceae* | -1 | 0 |
| *g__Acidibacter* | *g__Ilumatobacter* | -1 | 0 |
| *g__Acidibacter* | *g__Microbacterium* | -1 | 0 |
| *g__Aliihoeflea* | *g__Pseudomonas* | -1 | 0 |
| *g__Aliihoeflea* | *g__Luteolibacter* | -1 | 0 |
| *g__Aliihoeflea* | *g__Legionella* | 1 | 0 |
| *g__Bdellovibrio* | *g__Pseudomonas* | -1 | 0 |
| *g__Bdellovibrio* | *g__Luteolibacter* | -1 | 0 |
| *g__Bdellovibrio* | *g__Legionella* | 1 | 0 |
| *g__Bdellovibrio* | *g__Aliihoeflea* | 1 | 0 |
| *g__Bdellovibrio* | *g__Pedomicrobium* | 1 | 0 |
| *g__Bdellovibrio* | *g__norank_f__Sandaracinaceae* | 1 | 0 |
| *g__Bdellovibrio* | *g__Chryseobacterium* | 1 | 0 |
| *g__Blastomonas* | *g__Sphingopyxis* | 1 | 0 |
| *g__Brevundimonas* | *g__Bauldia* | 1 | 0 |

| | | | |
|---|---|---|---|
| *g__Brevundimonas* | *g__norank_f__Caldilineaceae* | 1 | 0 |
| *g__Chryseobacterium* | *g__Pseudomonas* | -1 | 0 |
| *g__Chryseobacterium* | *g__Luteolibacter* | -1 | 0 |
| *g__Chryseobacterium* | *g__Legionella* | 1 | 0 |
| *g__Chryseobacterium* | *g__Aliihoeflea* | 1 | 0 |
| *g__Chryseobacterium* | *g__Pedomicrobium* | 1 | 0 |
| *g__Chryseobacterium* | *g__norank_f__Sandaracinaceae* | 1 | 0 |
| *g__Exiguobacterium* | *g__Rhodobacter* | -1 | 0 |
| *g__Flavobacterium* | *g__Cytophaga* | -1 | 0 |
| *g__Hirschia* | *g__Pseudoxanthomonas* | -1 | 0 |
| *g__Hirschia* | *g__Mesorhizobium* | -1 | 0 |
| *g__Hirschia* | *g__norank_o__Microtrichales* | -1 | 0 |
| *g__Hirschia* | *g__norank_f__Azospirillaceae* | -1 | 0 |
| *g__Hirschia* | *g__Terrimonas* | 1 | 0 |
| *g__Hydrogenophaga* | *g__Flavihumibacter* | 1 | 0 |
| *g__Ilumatobacter* | *g__Hyphomonas* | 1 | 0 |
| *g__Ilumatobacter* | *g__norank_f__Microscillaceae* | 1 | 0 |
| *g__Legionella* | *g__Pseudomonas* | -1 | 0 |
| *g__Legionella* | *g__Luteolibacter* | -1 | 0 |
| *g__Luteolibacter* | *g__Pseudomonas* | 1 | 0 |
| *g__Mesorhizobium* | *g__Pseudoxanthomonas* | 1 | 0 |
| *g__Microbacterium* | *g__Hyphomonas* | 1 | 0 |
| *g__Microbacterium* | *g__norank_f__Microscillaceae* | 1 | 0 |
| *g__Microbacterium* | *g__Ilumatobacter* | 1 | 0 |
| *g__Mucilaginibacter* | *g__Sphingopyxis* | -1 | 0 |

| | | | |
|---|---|---|---|
| *g__Mucilaginibacter* | *g__Blastomonas* | -1 | 0 |
| *g__Mucilaginibacter* | *g__OLB13* | -1 | 0 |
| *g__Mucilaginibacter* | *g__norank_f__Ilumatobacteraceae* | -1 | 0 |
| *g__Mucilaginibacter* | *g__Pedobacter* | -1 | 0 |
| *g__norank_f__Azospirillaceae* | *g__Pseudoxanthomonas* | 1 | 0 |
| *g__norank_f__Azospirillaceae* | *g__Mesorhizobium* | 1 | 0 |
| *g__norank_f__Azospirillaceae* | *g__norank_o__Microtrichales* | 1 | 0 |
| *g__norank_f__Caldilineaceae* | *g__Bauldia* | 1 | 0 |
| *g__norank_f__Ilumatobacteraceae* | *g__Sphingopyxis* | 1 | 0 |
| *g__norank_f__Ilumatobacteraceae* | *g__Blastomonas* | 1 | 0 |
| *g__norank_f__Ilumatobacteraceae* | *g__OLB13* | 1 | 0 |
| *g__norank_f__Microscillaceae* | *g__Hyphomonas* | 1 | 0 |
| *g__norank_f__Sandaracinaceae* | *g__Pseudomonas* | -1 | 0 |
| *g__norank_f__Sandaracinaceae* | *g__Luteolibacter* | -1 | 0 |
| *g__norank_f__Sandaracinaceae* | *g__Legionella* | 1 | 0 |
| *g__norank_f__Sandaracinaceae* | *g__Aliihoeflea* | 1 | 0 |
| *g__norank_f__Sandaracinaceae* | *g__Pedomicrobium* | 1 | 0 |
| *g__norank_o__Microtrichales* | *g__Pseudoxanthomonas* | 1 | 0 |
| *g__norank_o__Microtrichales* | *g__Mesorhizobium* | 1 | 0 |
| *g__norank_o__Saccharimonadales* | *g__Actinoplanes* | 1 | 0 |
| *g__OLB13* | *g__Sphingopyxis* | 1 | 0 |
| *g__OLB13* | *g__Blastomonas* | 1 | 0 |
| *g__Pedobacter* | *g__Sphingopyxis* | 1 | 0 |
| *g__Pedobacter* | *g__Blastomonas* | 1 | 0 |
| *g__Pedobacter* | *g__OLB13* | 1 | 0 |

| | | | |
|---|---|---|---|
| *g__Pedobacter* | *g__norank_f__Ilumatobacteraceae* | 1 | 0 |
| *g__Pedomicrobium* | *g__Pseudomonas* | -1 | 0 |
| *g__Pedomicrobium* | *g__Luteolibacter* | -1 | 0 |
| *g__Pedomicrobium* | *g__Legionella* | 1 | 0 |
| *g__Pedomicrobium* | *g__Aliihoeflea* | 1 | 0 |
| *g__Planococcus* | *g__Acidovorax* | -1 | 0 |
| *g__Tahibacter* | *g__Hyphomicrobium* | 1 | 0 |
| *g__Terrimonas* | *g__Pseudoxanthomonas* | -1 | 0 |
| *g__Terrimonas* | *g__Mesorhizobium* | -1 | 0 |
| *g__Terrimonas* | *g__norank_o__Microtrichales* | -1 | 0 |
| *g__Terrimonas* | *g__norank_f__Azospirillaceae* | -1 | 0 |

**SUPPLEMENTAL FILES3**

**Table 1** Differential Metabolites between E+ and E- plants.

| Superclass | Common name | E+_1 | E+_2 | E+_3 | E+_4 | E-_1 | E-_2 | E-_3 | E-_4 |
|---|---|---|---|---|---|---|---|---|---|
| Alkaloids and derivatives | Ergometrine | 888513.9346 | 899878.6172 | 1547411.816 | 1475121.644 | 524646.6771 | 7678.6601 | 289174.629 | 5781.3957 |
| Alkaloids and derivatives | 6-allyl-8b-Carboxy-ergoline | 4983.3067 | 4309.2295 | 2177.7623 | 3155.735 | 4911.3321 | 2638.9564 | 4823.8357 | 843.8407 |
| Alkaloids and derivatives | Cinchonidine | 852.5812 | 0 | 0 | 0 | 887.128 | 0 | 822.7798 | 0 |
| Alkaloids and derivatives | Norajmaline | 172569.3544 | 195370.9313 | 235944.6588 | 159880.2766 | 42738.7498 | 0 | 24723.1722 | 0 |
| Benzenoids | Modafinil acid | 912270.5667 | 696300.0897 | 702014.6965 | 578110.038 | 180246.3421 | 227157.1425 | 244152.7765 | 290167.3961 |

| | | | | | | | | | |
|---|---|---|---|---|---|---|---|---|---|
| Benzenoids | Vibunazole | 1649226.317 | 1590244.869 | 2059048.607 | 1929756.643 | 226705.2887 | 374896.9765 | 208237.583 | 364843.7302 |
| Benzenoids | (2-Naphthalenyloxy)acetic acid | 48764.1127 | 53028.336 | 57971.2846 | 43707.5934 | 6704.8505 | 0 | 0 | 0 |
| Benzenoids | Desglymidodrine | 26624.4162 | 25400.0945 | 20674.2663 | 13668.0948 | 467.1716 | 2474.5137 | 873.6301 | 682.2461 |
| Benzenoids | Zingerone | 2424.619 | 1905.8703 | 9773.3429 | 9683.5271 | 1475.0413 | 9683.5271 | 1102.2405 | 599.1634 |
| Lipids and lipid-like molecules | Hydratopyrrhoxanthinol | 841293.917 | 790056.4942 | 1170513.392 | 937150.7321 | 0 | 46314.7865 | 0 | 27686.9174 |
| Lipids and lipid-like molecules | 17a-Ethynylestradiol | 107686.6529 | 102425.4341 | 199452.1773 | 244443.2853 | 29647.3577 | 0 | 22522.0449 | 0 |
| Lipids and lipid-like molecules | 17-alpha-ethinyl estradiol | 112091.7616 | 62654.9093 | 180218.5214 | 195675.2387 | 10647.4893 | 836.6822 | 5278.9621 | 892.5224 |
| Lipids and lipid-like molecules | Ethyl (4Z)-4,7-octadienoate | 5963.9946 | 4913.2511 | 7223.6807 | 6551.5211 | 1147.7664 | 0 | 0 | 481.515 |
| Lipids and lipid-like molecules | 13(S)-HpODE | 46928.6898 | 31779.1566 | 57629.4857 | 25144.2384 | 0 | 4990.9425 | 0 | 2946.8683 |
| Lipids and lipid-like molecules | 20-Hydroxy-leukotriene E4 | 0 | 1115.3755 | 14959.0825 | 9825.1881 | 0 | 0 | 0 | 0 |
| Lipids and lipid-like molecules | 12-oxo-20-dihydroxy-leukotriene B4 | 15137.2751 | 11682.2706 | 62310.6789 | 49702.3104 | 21944.8332 | 23728.7881 | 17032.9315 | 17004.6731 |
| Lipids and lipid-like molecules | Lauroyl diethanolamide | 257719.8806 | 245902.9213 | 54952.7476 | 58076.6051 | 14013.8152 | 0 | 15397.7794 | 37315.5063 |
| Lipids and lipid-like molecules | 9,10-Epoxyoctadecanoic acid | 67369.5832 | 43321.2941 | 3438.4739 | 3358.0938 | 1877.1023 | 0 | 1481.8272 | 2586.8543 |

| | | | | | | | | | |
|---|---|---|---|---|---|---|---|---|---|
| Lipids and lipid-like molecules | 8,12-Epoxy-4(15),7,11-eudesmatrien-1-one | 0 | 0 | 3244.3043 | 0 | 0 | 0 | 0 | 0 |
| Nucleosides, nucleotides, and analogues | S-Adenosylmethioninamine | 787772.5366 | 794699.4297 | 967340.7107 | 1079717.598 | 374399.5002 | 9700.6638 | 355680.8458 | 7839.4505 |
| Organic acids and derivatives | L-Dopa | 31647.8334 | 31683.509 | 25750.8735 | 18665.2602 | 20744.3593 | 19867.1269 | 13769.9175 | 27435.1075 |
| Organic acids and derivatives | O-Phosphotyrosine | 163466.5482 | 131770.8371 | 217088.4977 | 242913.7085 | 60573.223 | 0 | 58822.2621 | 0 |
| Organic acids and derivatives | O-(2-fluoroethyl)-l-tyrosine | 50959.0696 | 53766.1166 | 85277.9929 | 78289.1747 | 9360.9736 | 0 | 4053.2709 | 0 |
| Organic acids and derivatives | Tyrosyl-Leucine | 8687.0884 | 0 | 22172.3496 | 12268.3234 | 18447.8681 | 0 | 8793.8738 | 0 |
| Organic acids and derivatives | (-)-Fumigaclavine B | 99062.2459 | 104605.9512 | 88436.4549 | 69506.316 | 34714.9219 | 0 | 24335.1708 | 0 |
| Organic acids and derivatives | N1-(2-Methoxy-4-methylbenzyl)-n2-(2-(5-methylpyridin-2-yl)ethyl)oxalamide | 10693.8264 | 10359.4069 | 14048.0585 | 0 | 17874.873 | 0 | 14627.1116 | 0 |
| Organic acids and derivatives | Valyltryptophan | 579125.6864 | 646764.7549 | 1399581.642 | 1280180.161 | 250132.2505 | 2167.2319 | 129672.7157 | 0 |
| Organic acids and derivatives | Isoleucyl-Valine | 78554.1491 | 76576.2501 | 129933.0928 | 96570.6457 | 9951.7745 | 0 | 4470.9353 | 0 |
| Organic acids and derivatives | Ethylenediaminetetraacetic acid | 1598770.462 | 11502076.4 | 2357928.502 | 6834929.652 | 17052566.09 | 7957110.887 | 8520431.162 | 4926473.814 |
| Organic acids and derivatives | Ophthalmic acid | 136547.9126 | 141245.9736 | 88561.5485 | 69706.3864 | 0 | 0 | 947.6563 | 1754.2349 |
| Organic acids and derivatives | Gamma-Glutamylphenylalanine | 5983.0626 | 21322.1092 | 6147.1167 | 14038.5839 | 0 | 622.6558 | 0 | 690.154 |

| | | | | | | | | | |
|---|---|---|---|---|---|---|---|---|---|
| Organic acids and derivatives | Serylserine | 17630.9571 | 17289.6416 | 12164.2738 | 18016.091 | 0 | 1750.5007 | 611.4862 | 0 |
| Organic acids and derivatives | Gamma glutamyl ornithine | 250181.9349 | 219489.4862 | 177436.5548 | 186350.3711 | 16155.0547 | 18631.166 | 18671.7164 | 23111.5261 |
| Organic acids and derivatives | Glutaminylcysteine | 31587.5855 | 26583.4581 | 30056.9027 | 20959.2658 | 8992.9832 | 0 | 5231.1654 | 1088.1945 |
| Organic acids and derivatives | Lysylvaline | 24311.2397 | 24783.5455 | 65630.3744 | 30168.7675 | 7407.8107 | 2.2771 | 2921.326 | 0 |
| Organic acids and derivatives | Alanylhydroxyproline | 30650.9166 | 254524.6789 | 43064.4444 | 117520.6022 | 402179.2908 | 139681.3706 | 135527.0442 | 59727.5219 |
| Organic acids and derivatives | Tryptophyl-Tryptophan | 4730.2228 | 5725.2776 | 33081.9189 | 18129.4807 | 16752.4014 | 53870.11 | 11942.9674 | 49459.6105 |
| Organic acids and derivatives | L-Methionine | 243158.6717 | 282930.9164 | 490653.6887 | 272262.4385 | 24096.2753 | 19042.7114 | 4383.5824 | 52136.0061 |
| Organic acids and derivatives | Glutamylleucine | 3347.8016 | 5908.0188 | 9706.1635 | 6525.8491 | 1895.4283 | 2494.7123 | 0 | 8856.2767 |
| Organic acids and derivatives | Tryptophyl-Proline | 8687.0884 | 0 | 22172.3496 | 12268.3234 | 18447.8681 | 0 | 8793.8738 | 0 |
| Organic oxygen compounds | 6-(4-carboxyphenoxy)-3,4,5-trihydroxyoxane-2-carboxylic acid | 328229.6527 | 1961717.417 | 470510.8414 | 1097475.611 | 2568488.865 | 1460299.304 | 1329312.362 | 825545.2258 |
| Organic oxygen compounds | Dihydrozeatin-9-N-glucoside | 114365.2572 | 106915.2365 | 120932.5848 | 92706.686 | 35586.2232 | 0 | 44675.9203 | 0 |
| Organic oxygen compounds | N-Acetylgalactosamine 4-sulphate | 19329.3771 | 20477.094 | 821.7989 | 1726.6411 | 818.6724 | 1519.8992 | 0 | 9337.0646 |
| Organic oxygen compounds | 4-Hydroxycyclohexylcarboxylic acid | 6959.1704 | 9659.1684 | 12456.4468 | 16540.6361 | 0 | 0 | 0 | 0 |

| | | | | | | | | | |
|---|---|---|---|---|---|---|---|---|---|
| Organic oxygen compounds | Cis-Zeatin-7-N-glucoside | 69779.652 | 82935.1137 | 113978.8085 | 97677.9142 | 58117.865 | 0 | 50238.6732 | 0 |
| Organic oxygen compounds | 2-Aminoacetophenone | 82148.1278 | 78325.9301 | 43447.8057 | 17978.8433 | 37269.2617 | 0 | 21970.9383 | 0 |
| Organoheterocyclic compounds | (+/-)-2-Pentylthiazolidine | 6386.1574 | 10728.0209 | 20874.4074 | 12124.0157 | 1707.6159 | 0 | 945.8863 | 0 |
| Organoheterocyclic compounds | Hydroxylated N-acetyl desmethyl frovatriptan | 25375.4277 | 22590.0225 | 92190.3893 | 97154.5597 | 16649.4326 | 0 | 16325.8981 | 0 |
| Organoheterocyclic compounds | 6-(1-Hydroxyethyl)-2,2-dimethyl-2H-1-benzopyran | 82357.4569 | 78032.9817 | 98167.064 | 91472.4764 | 7542.4109 | 10977.0321 | 5407.0566 | 12547.5597 |
| Organoheterocyclic compounds | Hypoxanthine | 100755.6219 | 83980.535 | 138433.0546 | 91552.5567 | 14079.5986 | 8627.1586 | 8327.3347 | 18621.9935 |
| Organoheterocyclic compounds | 3-(3-Methylbutylidene)-1(3H)-isobenzofuranone | 82357.4569 | 78032.9817 | 98167.064 | 91472.4764 | 7542.4109 | 10977.0321 | 5407.0566 | 12547.5597 |
| Organoheterocyclic compounds | Xi-Anomuricine | 25726.133 | 24756.1154 | 26532.1259 | 17129.981 | 0 | 0 | 0 | 0 |
| Organoheterocyclic compounds | Indol-2-one | 1071486.746 | 949217.4907 | 1239231.903 | 1357377.395 | 573998.7367 | 13068.9419 | 556824.7115 | 11485.5065 |
| Organoheterocyclic compounds | (+/-)-Ribaline | 11067.3031 | 5623.1286 | 1204.79 | 0 | 2906.9227 | 0 | 0 | 0 |
| Organoheterocyclic compounds | 3-Indoleacetic Acid | 4461.2299 | 3166.5859 | 654.0223 | 337.9558 | 0 | 1306.2144 | 0 | 717.6118 |
| Phenylpropanoids and polyketides | Hordatine B | 204779.4953 | 186037.1706 | 305170.6449 | 264717.092 | 0 | 5411.7599 | 0 | 0 |
| Phenylpropanoids and polyketides | Alpha-Methylphenylalanine | 22978.1023 | 22880.7623 | 37029.3871 | 22509.3423 | 3135.6132 | 4948.9719 | 2577.0638 | 9152.3967 |

| | | | | | | | | | |
|---|---|---|---|---|---|---|---|---|---|
| Undefined | 3',6'-Dihydroxy-4,5-diaminospiro[isobenzofuran-1(3H),9'-[9H]xanthene]-3-one | 0 | 65154.8705 | 12636.4697 | 0 | 43000.8601 | 29527.9818 | 41094.9211 | 18654.4548 |
| Undefined | (S)-3-([1,1'-Biphenyl]-4-yl)-2-aminopropanoic acid | 8239.6727 | 7046.3041 | 20064.4218 | 12560.5903 | 4295.0456 | 0 | 0 | 0 |
| Undefined | His Ser Val | 10233.4652 | 10136.9828 | 7773.7238 | 0 | 9825.7509 | 0 | 4009.1626 | 0 |
| Undefined | Chanoclavine-I | 115210.5689 | 105357.8859 | 25613.3878 | 13882.3128 | 8157.5095 | 0 | 1289.3059 | 0 |
| Undefined | 4-[(3-hydroxy-7-azaspiro[3.5]nonan-7-yl)methyl]benzonitrile | 46710.9052 | 52957.8582 | 23151.4711 | 9488.4752 | 8520.1362 | 0 | 1087.029 | 0 |
| Undefined | 6,8-Dihydroxy-3-(10-hydroxyundecyl)-3,4-dihydro-1H-isochromen-1-one | 38466.4755 | 43430.7545 | 154078.9121 | 129918.2325 | 58154.7591 | 26944.1867 | 51909.3042 | 33194.5461 |
| Undefined | Indirubin-3'-monoxime | 53228.8136 | 45670.9891 | 60611.1559 | 66412.9847 | 15608.348 | 0 | 15206.0116 | 0 |
| Undefined | Isoscoparin 4'-glucoside | 0 | 0 | 244932.3106 | 58308.3048 | 457078.6629 | 117313.2542 | 107373.5673 | 16199.4069 |
| Undefined | Indarubicin | 16714.1782 | 18737.3169 | 45947.9154 | 41970.583 | 1607.1622 | 0 | 1726.0085 | 0 |
